# Human-lineage-specific genomic elements are associated with neurodegenerative disease and *APOE* transcript usage

**DOI:** 10.1038/s41467-021-22262-5

**Published:** 2021-04-06

**Authors:** Zhongbo Chen, David Zhang, Regina H. Reynolds, Emil K. Gustavsson, Sonia García-Ruiz, Karishma D’Sa, Aine Fairbrother-Browne, Jana Vandrovcova, International Parkinson’s Disease Genomics Consortium (IPDGC), Alastair J. Noyce, Rauan Kaiyrzhanov, Ben Middlehurst, Demis A. Kia, Manuela Tan, Huw R. Morris, Helene Plun-Favreau, Peter Holmans, Daniah Trabzuni, Jose Bras, John Quinn, Kin Y. Mok, Kerri J. Kinghorn, Kimberley Billingsley, Nicholas W. Wood, Patrick Lewis, Sebastian Schreglmann, Rita Guerreiro, Ruth Lovering, Lea R’Bibo, Claudia Manzoni, Mie Rizig, Sebastian Guelfi, Valentina Escott-Price, Viorica Chelban, Thomas Foltynie, Nigel Williams, Alexis Brice, Fabrice Danjou, Suzanne Lesage, Jean-Christophe Corvol, Maria Martinez, Claudia Schulte, Kathrin Brockmann, Javier Simón-Sánchez, Peter Heutink, Patrizia Rizzu, Manu Sharma, Thomas Gasser, Aude Nicolas, Mark R. Cookson, Sara Bandres-Ciga, Cornelis Blauwendraat, David W. Craig, Faraz Faghri, J. Raphael Gibbs, Dena G. Hernandez, Kendall Van Keuren-Jensen, Joshua M. Shulman, Hampton L. Leonard, Mike A. Nalls, Laurie Robak, Steven Lubbe, Steven Finkbeiner, Niccolo E. Mencacci, Codrin Lungu, Andrew B. Singleton, Sonja W. Scholz, Xylena Reed, Roy N. Alcalay, Ziv Gan-Or, Guy A. Rouleau, Lynne Krohn, Jacobus J. van Hilten, Johan Marinus, Astrid D. Adarmes-Gómez, Miquel Aguilar, Ignacio Alvarez, Victoria Alvarez, Francisco Javier Barrero, Jesús Alberto Bergareche Yarza, Inmaculada Bernal-Bernal, Marta Blazquez, Marta Bonilla-Toribio, Juan A. Botía, María Teresa Boungiorno, Dolores Buiza-Rueda, Ana Cámara, Fátima Carrillo, Mario Carrión-Claro, Debora Cerdan, Jordi Clarimón, Yaroslau Compta, Monica Diez-Fairen, Oriol Dols-Icardo, Jacinto Duarte, Raquel Duran, Francisco Escamilla-Sevilla, Mario Ezquerra, Cici Feliz, Manel Fernández, Rubén Fernández-Santiago, Ciara Garcia, Pedro García-Ruiz, Pilar Gómez-Garre, Maria Jose Gomez Heredia, Isabel Gonzalez-Aramburu, Ana Gorostidi Pagola, Janet Hoenicka, Jon Infante, Silvia Jesús, Adriano Jimenez-Escrig, Jaime Kulisevsky, Miguel A. Labrador-Espinosa, Jose Luis Lopez-Sendon, Adolfo López de Munain Arregui, Daniel Macias, Irene Martínez Torres, Juan Marín, Maria Jose Marti, Juan Carlos Martínez-Castrillo, Carlota Méndez-del-Barrio, Manuel Menéndez González, Marina Mata, Adolfo Mínguez, Pablo Mir, Elisabet Mondragon Rezola, Esteban Muñoz, Javier Pagonabarraga, Pau Pastor, Francisco Perez Errazquin, Teresa Periñán-Tocino, Javier Ruiz-Martínez, Clara Ruz, Antonio Sanchez Rodriguez, María Sierra, Esther Suarez-Sanmartin, Cesar Tabernero, Juan Pablo Tartari, Cristina Tejera-Parrado, Eduard Tolosa, Francesc Valldeoriola, Laura Vargas-González, Lydia Vela, Francisco Vives, Alexander Zimprich, Lasse Pihlstrom, Mathias Toft, Sulev Koks, Pille Taba, Sharon Hassin-Baer, John Hardy, Henry Houlden, Sarah A. Gagliano Taliun, Juan Botía, Mina Ryten

**Affiliations:** 1 Department of Neurodegenerative Disease, Queen Square Institute of Neurology, University College London (UCL), London, UK; 2 NIHR Great Ormond Street Hospital Biomedical Research Centre, University College London, London, UK; 3 Department of Genetics and Genomic Medicine, Great Ormond Street Institute of Child Health, University College London, London, UK; 4 Reta Lila Weston Institute, Queen Square Institute of Neurology, UCL, London, UK; 5 UK Dementia Research Institute, Queen Square Institute of Neurology, UCL, London, UK; 6 NIHR University College London Hospitals Biomedical Research Centre, London, UK; 7 Institute for Advanced Study, The Hong Kong University of Science and Technology, The Hong Kong University of Science and Technology, Hong Kong SAR, China; 8 Department of Neuromuscular Disease, Queen Square Institute of Neurology, UCL, London, UK; 9 Department of Medicine & Department of Neurosciences, Université de Montréal, Université de Montréal, Montréal, QC Canada; 10 Montréal Heart Institute, Montréal, Québec, Canada; 11 Departamento de Ingeniería de la Información y las Comunicaciones, Universidad de Murcia, Murcia, Spain; 12 Preventive Neurology Unit, Wolfson Institute of Preventive Medicine, Barts and the London School of Medicine and Dentistry, Queen Mary University of London, London, UK; 13 Department of Molecular Neuroscience, Queen Square Institute of Neurology, University College London (UCL), London, UK; 14 Institute of Translational Medicine, University of Liverpool, Liverpool, UK; 15 Department of Clinical and Movement Neuroscience, Queen Square Institute of Neurology, University College London (UCL), London, UK; 16 Biostatistics & Bioinformatics Unit, Institute of Psychological Medicine and Clinical Neuroscience, MRC Centre for Neuropsychiatric Genetics & Genomics, Cardiff, UK; 17 Department of Genetics, King Faisal Specialist Hospital and Research Centre, Riyadh, Saudi Arabia; 18 Institute of Healthy Ageing, University College London (UCL), London, UK; 19 University of Reading, Reading, UK; 20 UK Dementia Research Institute, University College London (UCL), London, UK; 21 Institute of Cardiovascular Science, University College London (UCL), London, UK; 22 MRC Centre for Neuropsychiatric Genetics and Genomics, Cardiff University School of Medicine, Cardiff, UK; 23 Institut du Cerveau et de la Moelle épinière, ICM, Inserm U 1127, CNRS, UMR 7225, Sorbonne Universités, UPMC University Paris 06, UMR S 1127, AP-HP, Pitié-Salpêtrière Hospital, Paris, France; 24 Institut du Cerveau et de la Moelle épinière, ICM, Inserm U 1127, CNRS, UMR 7225, Sorbonne Universités, UPMC University Paris 06, UMR S 1127, Centre d’Investigation Clinique Pitié Neurosciences CIC-1422, AP-HP, Pitié-Salpêtrière Hospital, Paris, France; 25 INSERM UMR 1220 and Paul Sabatier University, Toulouse, France; 26 Department for Neurodegenerative Diseases, Hertie Institute for Clinical Brain Research, German Center for Neurodegenerative Diseases, DZNE, German Center for Neurodegenerative Diseases, University of Tübingen, Tübingen, Germany; 27 Centre for Genetic Epidemiology, Institute for Clinical Epidemiology and Applied Biometry, University of Tubingen, Tübingen, Germany; 28 Laboratory of Neurogenetics, National Institute on Aging, Bethesda, MD USA; 29 National Institute of Neurological Disorders and Stroke, Bethesda, MD USA; 30 Department of Translational Genomics, Keck School of Medicine, University of Southern California, Los Angeles, CA USA; 31 Department of Computer Science, University of Illinois at Urbana-Champaign, Urbana, IL USA; 32 Neurogenomics Division, TGen, Phoenix, AZ USA; 33 Departments of Neurology, Neuroscience, and Molecular & Human Genetics, Baylor College of Medicine, Houston, TX USA; 34 Jan and Dan Duncan Neurological Research Institute, Texas Children’s Hospital, Houston, TX USA; 35 Data Tecnica International, Glen Echo, MD USA; 36 Ken and Ruth Davee Department of Neurology, Northwestern University Feinberg School of Medicine, Chicago, IL USA; 37 Department of Neurology and Physiology, University of California, San Francisco, CA USA; 38 Gladstone Institute of Neurological Disease, San Francisco, CA USA; 39 Taube/Koret Center for Neurodegenerative Disease Research, San Francisco, CA USA; 40 Northwestern University Feinberg School of Medicine, Chicago, IL USA; 41 National Institutes of Health Division of Clinical Research, NINDS, National Institutes of Health, Bethesda, MD USA; 42 Neurodegenerative Diseases Research Unit, National Institute of Neurological Disorders and Stroke, Bethesda, MD USA; 43 Department of Neurology, College of Physicians and Surgeons, Columbia University Medical Center, New York, NY USA; 44 Taub Institute for Research on Alzheimer’s Disease and the Aging Brain, College of Physicians and Surgeons, Columbia University Medical Center, New York, NY USA; 45 Montreal Neurological Institute and Hospital, Department of Neurology & Neurosurgery, Department of Human Genetics, McGill University, Montréal, QC Canada; 46 Department of Neurology, Leiden University Medical Center, Leiden, The Netherlands; 47 Instituto de Biomedicina de Sevilla Hospital Universitario Virgen del Rocío/CSIC/Universidad de Sevilla, Seville, Spain; 48 Fundació Docència I Recerca Mútua de Terrassa and Movement Disorders Unit, Department of Neurology, University Hospital Mutua de Terrassa, Terrassa, Barcelona Spain; 49 Hospital Universitario Central de Asturias, Oviedo, Spain; 50 Hospital Universitario San Cecilio de Granada, Universidad de Granada, Granada, Spain; 51 Instituto de Investigación Sanitaria Biodonostia, San Sebastián, Spain; 52 Hospital Clinic de Barcelona, Barcelona, Spain; 53 Hospital General de Segovia, Segovia, Spain; 54 Memory Unit, Department of Neurology, IIB Sant Pau, Hospital de la Santa Creu i Sant Pau, Universitat Autònoma de Barcelona, Barcelona, Spain; 55 Centro de Investigación Biomédica en Red en Enfermedades Neurodegenerativas, Madrid, Spain; 56 Centro de Investigacion Biomedica, Universidad de Granada, Granada, Spain; 57 Hospital Universitario Virgen de las Nieves, Instituto de Investigación Biosanitaria de Granada, Granada, Spain; 58 Hospital Universitario Marqués de Valdecilla-IDIVALS, Instituto de Investigación Sanitaria Fundación Jiménez Díaz, Madrid, Spain; 59 Hospital Universitario Virgen de la Victoria, Malaga, Spain; 60 Hospital Universitario Marqués de Valdecilla-IDIVAL, Santander, Spain; 61 Institut de Recerca Sant Joan de Déu, Terrassa, Barcelona Spain; 62 Hospital Universitario Ramón y Cajal, Madrid, Spain; 63 Movement Disorders Unit, Department of Neurology, IIB Sant Pau, Hospital de la Santa Creu i Sant Pau, Universitat Autònoma de Barcelona, Terrassa, Barcelona Spain; 64 Department of Neurology, Instituto de Investigación Sanitaria La Fe, Hospital Universitario y Politécnico La Fe, Valencia, Spain; 65 Department of Neurology, Hospital Universitario Infanta Sofía, Madrid, Spain; 66 Hospital Universitario Donostia, Instituto de Investigación Sanitaria Biodonostia, San Sebastián, Spain; 67 Fundació Docència i Recerca Mútua de Terrassa and Movement Disorders Unit, Department of Neurology, University Hospital Mutua de Terrassa, Terrassa, Barcelona Spain; 68 Department of Neurology, Hospital Universitario Fundación Alcorcón, Madrid, Spain; 69 Department of Neurology, Medical University of Vienna, Vienna, Austria; 70 Department of Neurology, Oslo University Hospital, Oslo, Norway; 71 Department of Pathophysiology, University of Tartu, Tartu, Estonia; 72 Department of Reproductive Biology, Estonian University of Life Sciences, Tartu, Estonia; 73 Department of Neurology and Neurosurgery, University of Tartu, Tartu, Estonia; 74 Movement Disorders Institute, Department of Neurology and Sagol Neuroscience Center, Chaim Sheba Medical Center, Ramat Gan, Israel; 75 Sackler Faculty of Medicine, Tel Aviv University, Tel Aviv, Israel

**Keywords:** Genetic variation, Alzheimer's disease

## Abstract

Knowledge of genomic features specific to the human lineage may provide insights into brain-related diseases. We leverage high-depth whole genome sequencing data to generate a combined annotation identifying regions simultaneously depleted for genetic variation (constrained regions) and poorly conserved across primates. We propose that these constrained, non-conserved regions (CNCRs) have been subject to human-specific purifying selection and are enriched for brain-specific elements. We find that CNCRs are depleted from protein-coding genes but enriched within lncRNAs. We demonstrate that per-SNP heritability of a range of brain-relevant phenotypes are enriched within CNCRs. We find that genes implicated in neurological diseases have high CNCR density, including *APOE*, highlighting an unannotated intron-3 retention event. Using human brain RNA-sequencing data, we show the intron-3-retaining transcript to be more abundant in Alzheimer’s disease with more severe tau and amyloid pathological burden. Thus, we demonstrate potential association of human-lineage-specific sequences in brain development and neurological disease.

## Abstract (web summary)

Knowledge of genomic features specific to humans may be important for understanding disease. Here the authors demonstrate a potential role for these human-lineage-specific sequences in brain development and neurological disease.

## Introduction

Humans are perceived to be particularly vulnerable to neurodegenerative disorders relative to other primates on both a pathological and phenotypic level^1–5^. This is exemplified in Alzheimer’s disease, in which a similar phenotype is not seen in ageing non-human primates, nor are the characteristic neurofibrillary tangles on pathological examination^1,6^. Likewise, Parkinson’s disease does not naturally occur in non-human primates, whose motor deficits do not respond to levodopa administration and a Lewy body pathological burden is not present^5,7^. This has led to the hypothesis that the same evolutionary changes driving encephalisation which have steered the development of characteristic human features may predispose to disorders that affect the brain^2,5,6^. In the case of Alzheimer’s disease, it is postulated that the accelerated evolution of intelligence, brain size and lifespan predisposes to selective advantages, which in later life have deleterious effects on cognition through the very same pathways^8^. Therefore, identifying the genomic changes unique to the human lineage may not only provide insights into the evolution of human-lineage-specific phenotypic features but also into the pathophysiology underlying uniquely human diseases.

Previous studies attempting to identify human-lineage-specific variation and functional elements in the human genome have focused on genomic conservation as calculated by aligning and comparing genomes across species. But, conservation measures alone do not fully identify regions with evidence of human-specific purifying selection. This is because a large part of the genome is evolving neutrally and sufficient phylogenetic distance is required to detect these changes^9^. Furthermore, alignment methods do not reliably detect substitutions that preserve function^9^. Conversely, some genes such as those implicated in immune system function may be subject to rapid evolutionary turnover even among closely related species^9^. For these reasons, analysing conservation alone has limited capacity to capture human-specific genomic elements^9^.

The increasing availability of whole-genome sequencing (WGS) has opened new opportunities to address this issue. Using intra-species whole-genome comparisons^10,11^, we are better able to appreciate sequence differences between individuals of the same species, and identify genomic regions in humans containing significantly fewer genetic variants than expected by chance, designated as constrained genomic regions. This form of analysis, which is based on the assumption that most selection is negative or purifying (i.e. those that remove new deleterious mutations), has been crucial for classification of exonic variation and attribution of pathogenicity^12^. However, many genomic regions would be expected to be both constrained and conserved; such regions have been maintained by natural selection across species, including humans. This means that metrics reflecting constraint alone cannot identify human-specific elements as the same regions could also be conserved in other species.

This has led previous analyses to combine these metrics of sequence constraint and conservation to identify genomic regions with evidence for human-specific selection^13,14^. Ward and Kellis successfully applied this approach to demonstrate that a range of transcribed and regulatory non-conserved elements showed evidence of lineage-specific purifying selection^14^. However, this analysis was limited by the availability of WGS data and metrics on human genetic variation were derived from the 1000 Genomes pilot data, which sequenced with only two to six times coverage^15^. Advances in sequencing technology have increased the feasibility of deep sequencing of human populations leading to a much more detailed understanding of genetic variation between humans^10^. In fact, the recent sequencing of the genomes of 10,545 human individuals at a coverage of 30–40 times identified 150 million single-nucleotide variants of which 54.7% had not been reported in dbSNP^16^ or the most recent phase 3 of the 1000 Genomes Project^17^. The availability of this information has already enabled more accurate identification of relatively constrained regions of the genome, which has led to the development of the context-dependent tolerance score (CDTS)^11^. CDTS is derived from estimating how the observed genetic variation compares to the propensity of a nucleotide to vary depending on its surrounding context using the high-resolution profiles determined from deep sequencing data^11^. Yet, this information has not been combined directly with improved conservation data to identify regions with evidence for human-specific selection.

In this study, we make full use of these resources to develop a novel, granular genomic annotation which efficiently captures information on intra-species constraint and inter-species conservation simultaneously and identifies constrained, non-conserved regions (CNCRs). We use this annotation to test the hypothesis that CNCRs are not only specific to the human lineage, but given the encephalisation of humans, that CNCRs will be enriched within brain-specific functional and regulatory elements as well as risk loci for neurological disease. We show that these regions are enriched for SNP heritability for a range of neurological and psychiatric phenotypes. Furthermore, by calculating CNCR density within the boundaries of known genes, we develop a gene-based metric of human-specific constraint. This analysis highlights *APOE* and leads to the identification of an intron-3 retaining transcript of *APOE*, the usage of which is correlated with Alzheimer’s disease pathology and *APOE*-ε4 status. This approach provides direct support for the role of human-specific CNCRs in brain development and complex neurological phenotypes.

## Results

### Genomic regions with high constraint, but not conservation, were enriched for regulatory, non-coding genomic features

**Fig. 1:**
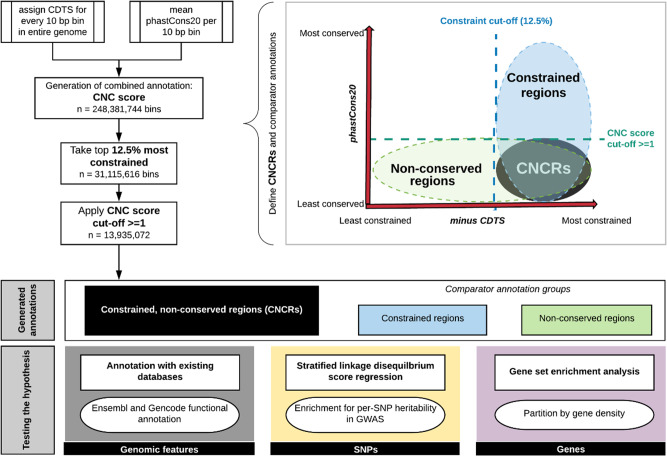
Workflow of study and schematic demonstration of annotation groups. The workflow depicts the processes involved in creation of the annotation with set parameters for each of the three groups of annotations generated and the processes involved in hypothesis-testing. CNC scores: constrained, non-conserved scores; CNCRs: constrained, non-conserved regions: CNCRs are defined as genomic regions that were first among the 12.5% most constrained, then with a CNC score of ≥1 (i.e. a twofold higher ranking in constraint than conservation). Constrained regions are defined as the regions within the 12.5% most constrained of the genome irrespective of conservation score. Non-conserved regions are defined as relatively non-conserved genomic regions with a conservation rank determined by the rank of the first quartile phastCons20 score at a CNC score of 1 (rank ≤ 25,623,592) (irrespective of constraint score). CDTS is the context-dependent tolerance score. Minus CDTS score is used as a lower score of CDTS corresponds to a more constrained region.

**Fig. 2:**
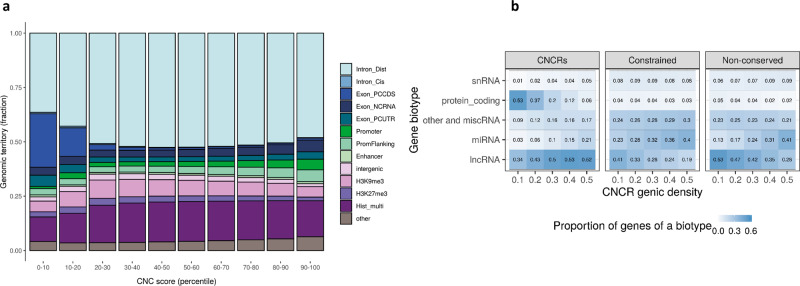
Genomic territory and biotype proportions of constrained, non-conserved regions. Composition of the constrained genome, partitioned by constrained, non-conserved (CNC) scores (**a**) and proportion of biotypes of genes in our annotation (constrained, non-conserved regions: CNCRs) and in the comparator annotations (constrained regions and non-conserved regions) (**b**). The description for each genomic feature is shown in Supplementary Table 1. The barplot in **a** shows the genomic features for the 12.5% most constrained regions with CNC scores partitioned by decile, such that the highest decile (90–100) represents the most constrained and least conserved regions. Description of gene biotypes in **b** is taken from Ensembl^42^. The heatmap demonstrates the proportion of genes of a certain biotype within the three separate annotations within each genic CNCR density cut-off. CNCR density is defined as the proportion of CNCRs within a gene taking into account the gene size. Protein coding is defined by a gene that contains an open reading frame. The subclassified components of long non-coding RNA (lncRNA) found in the annotations are: Antisense—has transcripts that overlap the genomic span (i.e. exon or introns) of a protein-coding locus on the opposite strand; lincRNA (long interspersed ncRNA)—has transcripts that are long intergenic non-coding RNA locus with a length > 200 bp; non-coding RNA is further subclassified into miRNA (microRNA); siRNA (small interfering RNA); snRNA (small nuclear RNA) and miscellaneous RNA (includes snoRNA (small nucleolar RNA) and tRNA (transfer RNA)). Pseudogenes are similar to known proteins but contain a frameshift and/or stop codon(s) which disrupts the open reading frame. These can be classified into processed pseudogene—a pseudogene that lacks introns and is thought to arise from reverse transcription of mRNA followed by reinsertion of DNA into the genome and unprocessed pseudogene—a pseudogene that can contain introns since produced by gene duplication.

CNC scores, which combine information from CDTS and phastCons20, were used to capture evidence of disparity between constraint and conservation within a genomic region (Fig. 1). We investigated the relationship between CNC scores and known genomic features within the most constrained portion of the genome (top 12.5%). This analysis demonstrated clear patterns of enhancement and depletion for genomic elements across CNC scores, which significantly differed from similar analyses performed using constraint metrics alone^11^ (Fig. 2a). Among constrained genomic regions with the highest CNC scores (90 to 100 decile, signifying high constraint but low conservation), we saw a depletion for coding elements of 27-fold relative to genomic regions with the lowest CNC scores (chi-squared *p* < 2.2 × 10^−16^). This contrasts with the pattern using constraint metrics alone where the most constrained genomic regions are highly enriched for coding exons^11^. On the other hand, promoter, promoter-flanking and non-coding RNA features were overrepresented in the highest compared to the lowest CNC deciles by 4.7- (chi-squared *p* < 2.2 × 10^−16^), 1.9- (chi-squared *p* < 2.2 × 10^−16^) and 1.5-fold (chi-squared *p* < 2.2 × 10^−16^) respectively. Thus, genomic regions with high CNC scores are enriched for regulatory, non-coding genomic features.

### Genes with the highest density of CNCRs are enriched for long non-coding RNA

Next, we applied a CNC score cut-off of ≥1 (signifying a twofold higher ranking in constraint than conservation) to the 12.5% most constrained genomic regions to define a set of genomic regions that were constrained, but not conserved (termed CNCRs). We wanted to investigate whether CNCRs could be used to identify specific genes of interest. With this in mind, we used CNCR density, the proportion of CNCRs within a gene (defined in Supplementary Fig. 1), to identify gene sets which might be expected to contribute most to human-specific phenotypes. Consistent with the findings above, we found that as the CNCR density threshold was increased to define the gene sets of interest, there was a marked reduction in the proportion of protein-coding genes (β-coefficient between proportion and CNCR density = −1.061 and false discovery rate (FDR)-corrected *p* = 0.00162), and an increase in the proportion of long non-coding RNA (lncRNA, β-coefficient 0.385 and FDR-corrected*p* = 0.0161) and microRNA-encoding genes (miRNA, β-coefficient 0.394 and FDR-corrected *p* = 0.00116) (Fig. 2b). Interestingly, this relationship was not clearly observed when considering unprocessed snRNA and other RNAs (Fig. 2b). In order to determine whether the relationship between CNCR density and gene biotype was driven by sequence constraint or conservation, we also generated comparator gene lists based on constrained-only and non-conserved regions alone. Importantly, lncRNA and protein-coding gene proportions do not follow the same directionality with increasing density when constraint or non-conservation alone is considered (Fig. 2b). Thus, this analysis highlighted the specific importance of lncRNAs as compared to other classes of non-coding RNAs in driving human-specific patterns of gene expression.

### Significant enrichment of heritability for neurologically relevant phenotypes

**Fig. 3:**
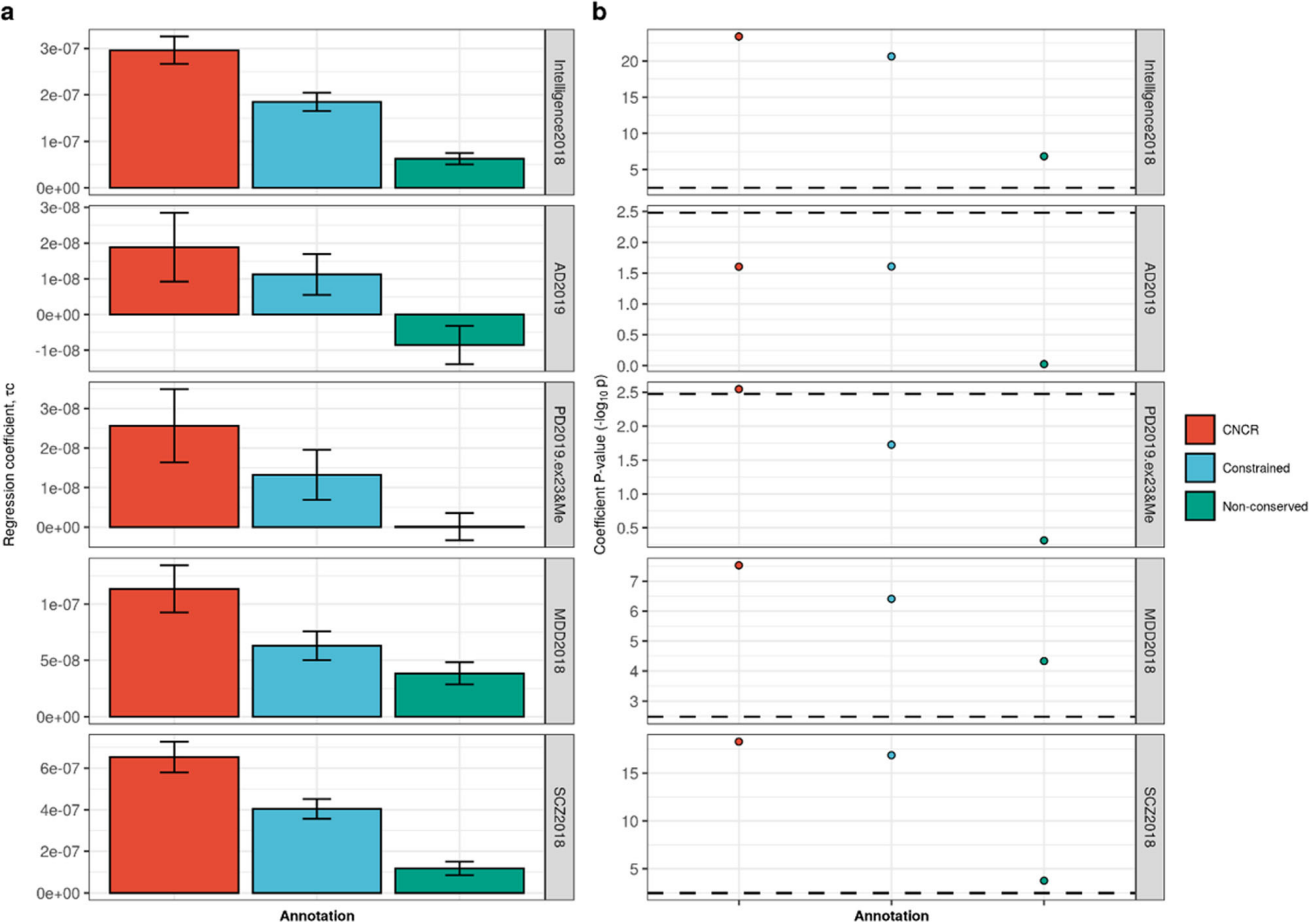
Stratified-linkage disequilibrium score regression (s-LDSC) analysis across five traits comparing constrained, non-conserved regions (CNCRs) with its constituent constrained and non-conserved annotations. **a** The regression coefficient. **b** The regression coefficient −log_10_(*p* value) with the dotted line showing the Bonferroni-corrected *p* value of 0.00333 for 15 conditions. GWASs were as follows: Intelligence2019: intelligence test performance GWAS, AD2018: Alzheimer’s disease GWAS, PD2019.ex23&Me: Parkinson’s disease GWAS without 23&Me data, MDD2018: major depressive disorders GWAS and SCZ2018: schizophrenia GWAS (Supplementary Table 2).

Given the enrichment of regulatory features within genomic regions with a high CNC score, we postulated that such regions could also be enriched for disease risk. In order to study this, we investigated CNCRs for evidence of enriched heritability for a range of complex neurologically relevant phenotypes (Supplementary Table 4). After Bonferroni correction for multiple testing, we found that CNCRs exhibited significant enrichment in heritability for intelligence test performance (coefficient *p* = 4.19 × 10^−24^); Parkinson’s disease (coefficient *p* = 4.65 × 10^−5^); major depressive disorder (coefficient *p* = 2.95 × 10^−8^) and schizophrenia (coefficient *p* = 5.26 × 10^−19^), but not for Alzheimer’s disease (Fig. 3). While a significant enrichment in heritability for intelligence test performance, major depressive disorder and schizophrenia were also observed in the constrained regions alone (and to a lesser extent, non-conserved regions), we noted that the regression coefficient for CNCRs was at least twofold larger for the CNCR annotation compared to the constrained annotation (Supplementary Table 4). Similarly, significant enrichment in heritability for Parkinson’s disease was only observed in CNCRs. SNP heritability for Alzheimer’s disease did not show significant enrichment although there was a trend for enrichment in terms of the regression coefficient and coefficient *p* value within CNCRs. Thus, by combining metrics for both constraint and conservation in our annotation, we derived an independent annotation that shows a higher level of enrichment in heritability for neurologically related phenotypes than annotations based on constraint or conservation alone.

### The proportion of enriched gene sets with neurologically related GO terms increases in genes with the highest density of CNCRs

**Fig. 4:**
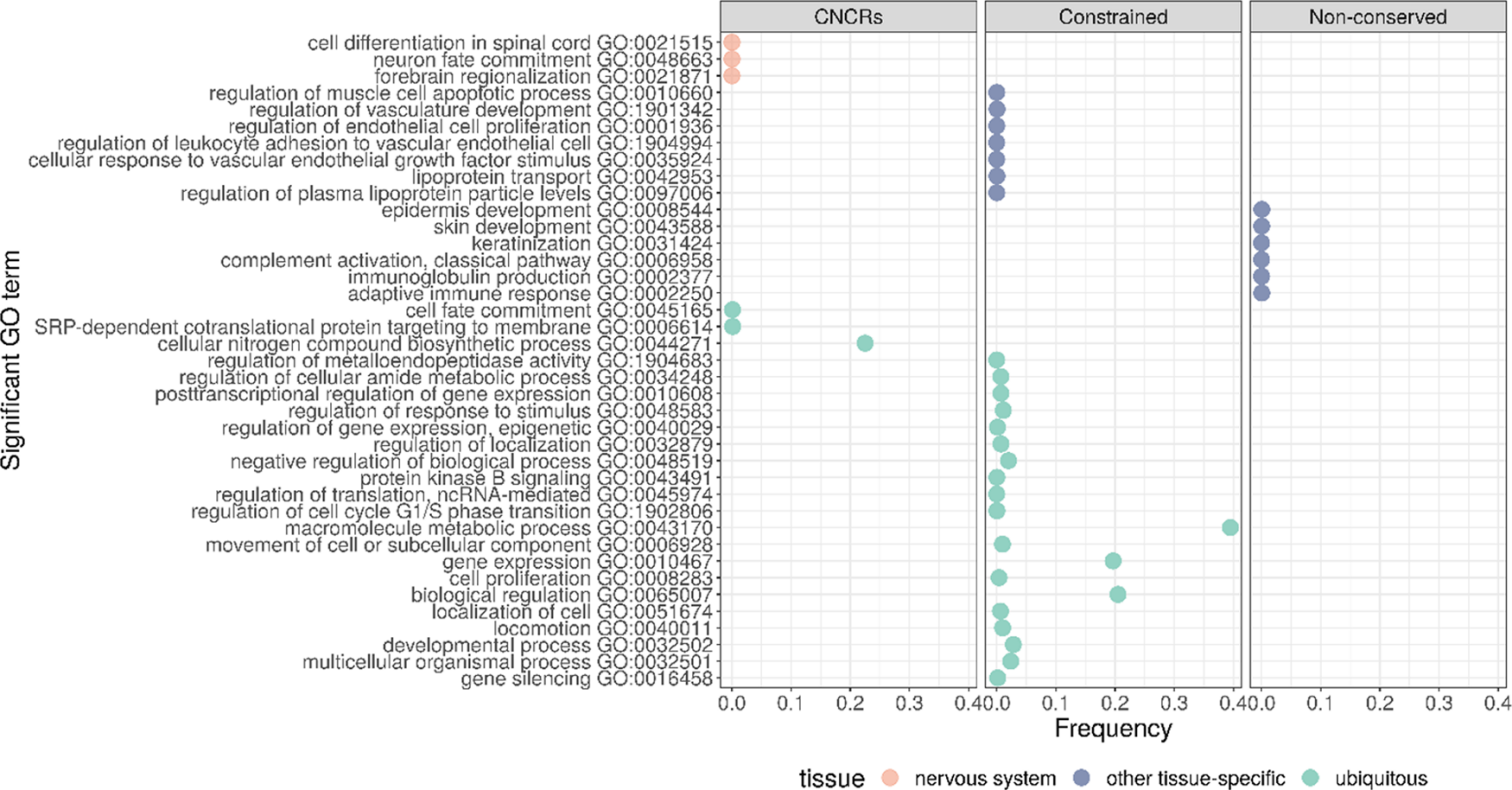
Summarised enriched gene sets for terms specific for neurological gene sets, other non-neurological-specific tissues and non-tissue-specific as defined by Gene Ontology (GO). Plot comparing annotation of interest (CNCRs) and comparator annotations which only use constraint or non-conserved metrics. Frequency, derived from REViGO^49^, represents the percentage of human proteins in UniProt which were annotated with a GO term, i.e. a higher frequency denotes a more general term.

To investigate these findings further, we defined gene sets based on their CNCR density (the proportion of CNCRs within a gene) and analysed their GO term enrichment. We assessed gene sets defined across a range of CNCR densities (>0.0 to ≥0.5 at 0.1 increments). We found that the proportion of neurologically associated GO terms with significant enrichments (g:SCS-corrected *p* < 0.05) increased among gene sets with increasing CNCR gene densities (Supplementary Fig. 2). Importantly, a similar analysis of gene sets defined by constraint alone or non-conservation alone did not contain any neurologically enriched GO terms (Fig. 4). We identified the gene set with the highest proportion of nervous system-related terms at a CNCR genic density of 0.3 (Supplementary Fig. 2). The only GO terms specific to a tissue process were related to the nervous system (Fig. 4, Supplementary Table 5) and spanned terms such as neuronal development (GO:0048663, corrected *p* = 5.46 × 10^−7^) and spinal cord differentiation (GO:0021515, corrected *p* = 3.64 × 10^−7^). The remaining significantly enriched GO terms related to ubiquitous processes including protein targeting (GO:0045047, *p* = 9.93 × 10^−4^) and DNA binding (GO:0043565, *p* = 4.81 × 10^−4^). Of note, analysis of gene sets defined on the basis of constraint alone revealed no enrichment of neurologically associated terms, but instead significant enrichment of vascular system-related GO terms (GO:0048514 blood vessel morphogenesis, corrected *p* = 3.96 × 10^−37^ and GO:0072358 cardiovascular system development, *p* = 8.53 × 10^−36^). As might be expected based on the rapid and potentially divergent evolutionary pressures, the analysis of gene sets defined on the basis of non-conservation alone demonstrated the significant enrichment of immune and skin-related GO terms (GO:0002250 adaptive immune response, *p* = 4.02 × 10^−10^ and GO:0043588 skin development, *p* = 2.33 × 10^−4^). Taken together, these results demonstrate that using CNCR density, genes important in nervous system development and implicated in neurological disease can be identified.

### CNCR annotation highlights an intron-3 retaining transcript of *APOE*

Next, we investigated the distribution of CNCR density across Mendelian genes associated with a neurological phenotypes (as defined within Online Mendelian Inheritance in Man (OMIM)^18^) and genes implicated in complex brain-relevant phenotypes (as defined within Systematic Target OPportunity assessment by Genetic Association Predictions (STOPGAP)^19^). We noted that the median CNCR density was significantly higher in OMIM genes with a neurological phenotype compared to all other genes (median CNCR density of neurological OMIM genes = 0.0924, IQR = 0.0567 − 0.143; median CNCR density of all other genes = 0.083, IQR = 0.043 − 0.153; Wilcoxon rank sum test *p* = 1.8 × 10^−6^). While genes associated with complex brain-relevant phenotypes did not have a significantly higher CNCR density when compared to all other genes, we still identified 31 genes with a CNCR density of greater than 0.2 and 7 genes with a CNCR density of greater than 0.3 (*APOE, PHOX2B, SSTR1, HCFC1, HAPLN4, CENPM* and *IQCF5*). Of these genes, *APOE* had the highest CNCR density with a value of 0.552.

**Fig. 5:**
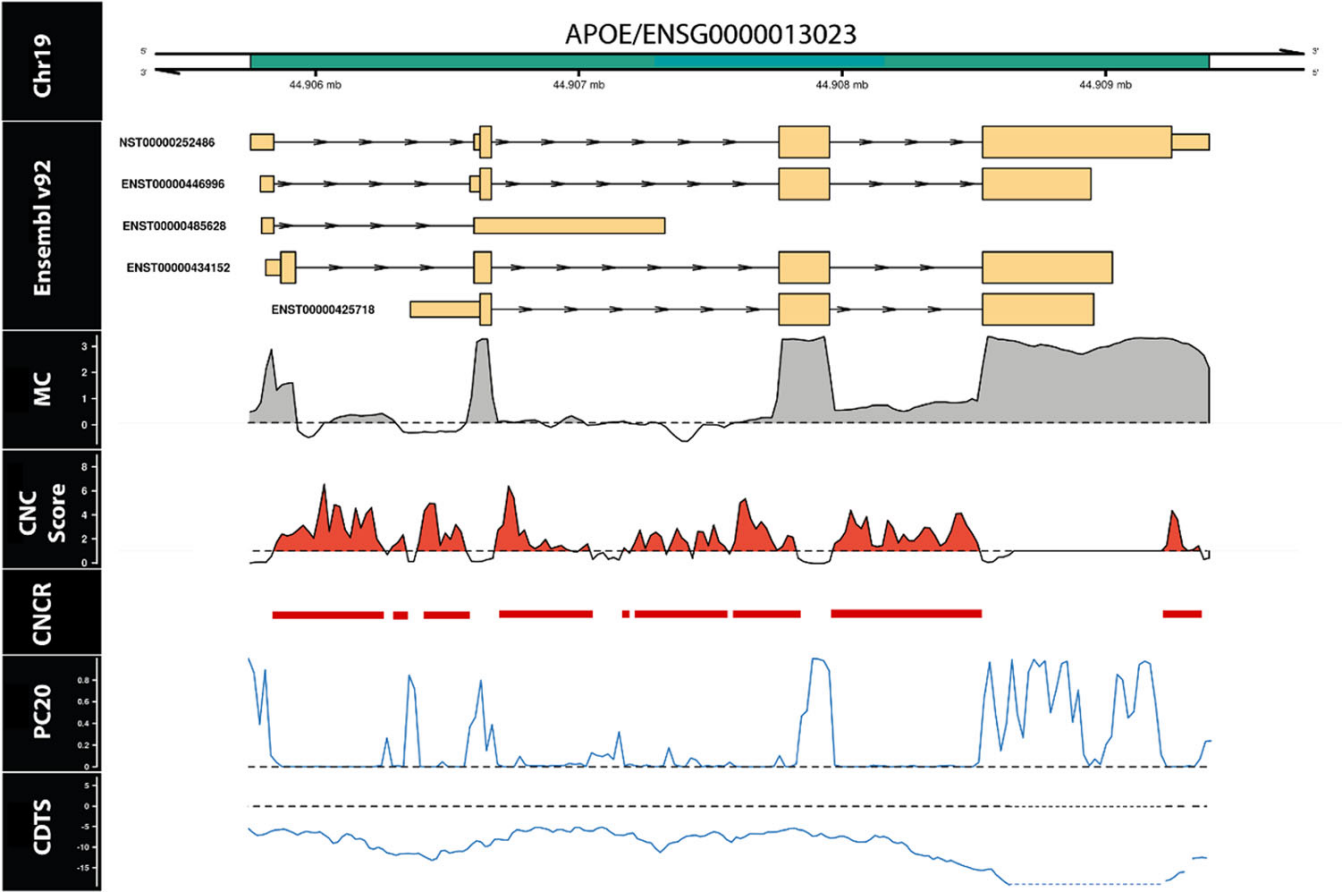
Annotation with constrained, non-conserved regions (CNCRs) is highly granular and shows *APOE* to have a high density of CNCRs throughout its length especially in association with an intron-3 retention event in the human hippocampus. The first track represents the genomic location of *APOE* within chromosome 19. The second track shows the known transcripts, currently within annotation in Ensembl v.92. The mean coverage (MC) (log_10_ scale) in the hippocampus shown here is greater than zero (denoted by the grey shaded area) across intron-3 highlighting an intron-3 retention event (mean coverage data derived from GTEx v.7). In the fourth track, CNC scores above the black dashed line and shaded in red fulfil criteria for a constrained, non-conserved region (CNCR) are shown. The intron-3 retention event has the highest CNCR density among all intronic regions of *APOE*. The fifth track labelled “CNCR” depicts regions fulfilling criteria for CNCR. PC20 represents the mean phastCons20 score. The black dashed line within this track represents a mean phastCons20 score of 0. CDTS represents the context-dependent tolerance score as a measure of constraint with the black dashed line showing a CDTS of 0. Within the CDTS track, the blue dotted line represents a region with no CDTS annotation.

Given the high CNCR density of *APOE*, its importance as a disease locus for Alzheimer’s disease and other neurodegenerative diseases^20^ and the long-standing interest in its lineage specificity^8,21^ (specifically the differences in the ɛ4 allele between humans and non-human primates^1^), we chose to focus on this gene to further validate our annotation. We tested whether intragenic analysis of *APOE* could identify specific regions or transcripts of interest. We compared CNCR density, constraint and conservation scores across the length of the gene showing that CNCRs provide a highly granular annotation (Fig. 5). Using this approach, we identified the region with the highest CNCR density in *APOE* to be within intron-3 (Supplementary Fig. 3), coinciding with the annotated region’s boundaries. Furthermore, the intron-3 region had a higher coverage compared to introns 1 and 2 based on the mean coverage provided by Genotype-Tissue Expression Consortium (GTEx) hippocampal tissue indicating that this is likely to represent an intron retention event (Supplementary Fig. 3E). This coverage was calculated as the mean across all GTEx samples normalized to a target library size of 40 million 100 base pair (bp) reads (mean coverage seen in Fig. 5)^22^. Thus, in conjunction with the highest intragenic CNCR density localised to intron-3, these coverage data provided further justification for our analysis of the intron-3 retention event.

Although no intron-3 retaining transcript is currently annotated in Refseq and Ensembl, an intron-3 retention event has previously been reported and implicated in the regulation of *APOE* expression^21,23,24^. To validate the existence of this transcript, we performed Sanger sequencing of poly-A-selected RNA derived from human hippocampal tissue. This demonstrated the existence of a transcript containing the full-length intron-3 sequence was flanked by both exon 3 and exon 4 (Supplementary Fig. 4a). Using a non-RT control, we showed that this could not be explained by genomic DNA (gDNA) contamination (Supplementary Fig. 4b).

We noted that transcripts retaining intron-3 of *APOE* are unannotated by Ensembl for humans and chimpanzees (Supplementary Fig. 5a). However, reads aligning to intron-3 were observed for the three human transcriptomes but do not occur in abundance for the three chimpanzee samples (Supplementary Fig. 5b, c). We also noted that there was a trend for lower expression of intron-3 within chimpanzees compared to humans (Supplementary Fig. 6). However, this analysis was limited by the inherently small sample sizes and so comparing the coverage of intron-3 normalised for the total coverage of *APOE* within the samples did not identify statistically significant differences in the expression of intron-3 across species. Nonetheless, these data would suggest that intron-3 retaining transcripts are more commonly expressed in humans, and likely to be largely absent in chimpanzees.

**Fig. 6:**
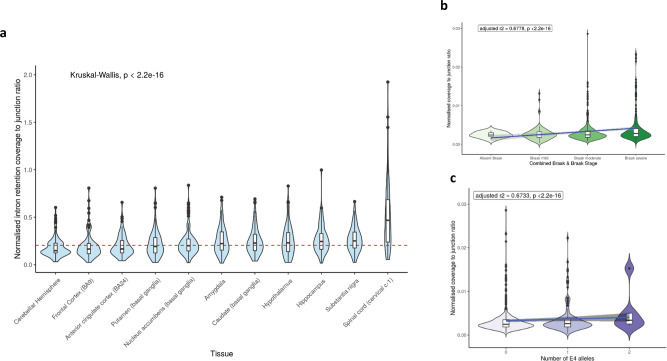
Quantification of *APOE* intron-3 retaining transcript usage. Quantification of intron retention usage by its normalised coverage to junction ratio across brain tissues within GTEx (**a**). Normalised coverage to junction ratio of the *APOE* intron-3 retention event in bulk RNA-sequencing data of post-mortem dorsolateral prefrontal cortex tissue samples from 634 individuals recruited within ROSMAP studies across Braak and Braak staging (**b**) and *APOE* ɛ4 allele status (**c**). In **a**, red dashed horizontal line presents the median normalised intron retention coverage to junction ratio within central nervous system tissues in GTEx. Number of samples within each of the tissue groups was as follows: amygdala—72; anterior cingulate cortex—84; caudate—117, cerebellar hemisphere—105; frontal cortex—108; hippocampus—94; hypothalamus—96; nucleus accumbens—113; putamen—97; spinal cord—71; substantia nigra—63. The Kruskal–Wallis *p* value show results from comparison of the differences in the normalised intron retention coverage to junction ratio between the different brain regions with pairwise regions comparisons shown in Supplementary Table 6. In **b** and **c**, the blue line represents the linear regression fit with the grey shaded area representing ± 95% confidence interval. Braak and Braak staging is a measure of severity of neurofibrillary tangle based on location. To improve the power of the study, we merged Braak and Braak stages I and II to “Braak mild stage”, Braak and Braak stages III and IV to “Braak moderate” and Braak and Braak stages V and VI to indicate “Braak severe” stage. For number of *APOE* ɛ4 alleles, a heterozygous state is represented by “1” and homozygous state by “2”.

In order to obtain further insights into the biological significance of the intron-3 retaining *APOE* transcript, we leveraged publicly available RNA-sequencing data covering 11 regions of the human central nervous system provided by the GTEx v.7^25^. Using an annotation-independent approach to identify genomic regions producing stable transcripts^26,27^, we identified a region of significant expression encompassing intron-3 of *APOE* and the flanking coding exons in all brain tissues (Fig. 6a). These data not only support the existence of an intron-3 retaining *APOE* transcript that is not entirely attributable to pre-mRNA transcripts or driven by background noise in sequencing but also provide a means of estimating its usage across the human brain.

Thus, in order to compare usage of this transcript across different CNS regions, we calculated the ratio of normalised intron-3 expression (a measure of intron-3 retaining transcripts) to the normalised expression of exon 3/exon 4 spanning reads (a measure of transcripts splicing out intron-3). We see that there is evidence of the usage of the intron-3 retaining *APOE* transcript in all central nervous system regions from GTEx data (Fig. 6a). However, there are also significant differences among brain regions (Kruskal–Wallis *p* < 2.2 × 10^−16^) (Supplementary Table 6**:** pairwise Wilcoxon rank sum test *p* values) with the usage of the intron-3 retaining event being highest in the spinal cord, substantia nigra and hippocampus (Fig. 6a).

In summary, we confirmed the existence of an unannotated human-specific non-coding transcript of *APOE* and identified differential usage of this transcript across the human brain. In this way, we demonstrated the utility of combining CNC scores with transcriptomic data, which we have made easier through the visualisation platform vizER (https://snca.atica.um.es/browser/app/vizER). Furthermore, this direct visualisation allows identification of isolated intragenic regions of functional importance in genes with highly variable CNCR density.

### Usage of the intron-3 retaining transcript of *APOE* correlates with Alzheimer’s disease pathology and *APOE* genotype

We noted that among the brain tissues with the highest usage of the intron-3 retaining transcript of *APOE* are those that show selective vulnerability for neurodegeneration, namely the hippocampus in the context of Alzheimer’s disease, the substantia nigra in the context of Parkinson’s disease and the spinal cord in the context of amyotrophic lateral sclerosis (pairwise comparisons between brain regions shown in Supplementary Table 6).

Given that *APOE* is one of the most important genetic risk factors for Alzheimer’s disease, we leveraged publicly available RNA-sequencing data from the Religious Orders Study and Memory and Aging Project (ROSMAP) studies to quantify the usage of the intron-3 retaining transcript of *APOE* in post-mortem dorsolateral prefrontal cortex brain tissue derived from individuals with Alzheimer’s disease (*n* = 222) and mild cognitive impairment (MCI) (*n* = 158) compared to control individuals (defined as the final clinical diagnosis blinded to pathological findings, *n* = 202). Prior to our analyses, we assessed the impact of batch effects within this dataset. After finding that our analyses were robust to the removal of an outlying batch (batch 7, Supplementary Fig. 7), we incorporated all batches into the analyses. Using this approach, we found that the proportion of the intron-3-retaining transcript was higher (*p* < 2.2 × 10^−16^) in dorsolateral prefrontal cortex tissue from individuals with clinically diagnosed Alzheimer’s disease and MCI patients versus control participants. Partitioning this further on the basis of pathology, we see an increase in intron-3 retaining transcript usage with more severe Braak and Braak pathology for neurofibrillary tangles (adjusted *r*^2^ 0.678, *p* < 2.2 × 10^−16^) (Fig. 6b). Consistent with these findings, we also found a significant increase in transcript usage with higher amyloid plaque pathology as defined using CERAD staging (adjusted *r*^2^ 0.673, *p* < 2.2 × 10^−16^). Finally, we investigated the relationship between presence of the ε4 allele in *APOE* and usage of the intron-3 retaining transcript. We found a significant positive correlation between ε4 allele load and the proportion of intron-3 retaining transcript (adjusted *r*^2^ 0.673, *p* < 2.2 × 10^−16^) (Fig. 6c). This association remained significant after partitioning *APOE*-ε4 status by disease and accounting for tau and amyloid burden, showing that this association is likely to be independent of disease state.

Taken together, these findings could suggest that usage of the intron-3 retaining transcript may be regulated by *APOE*-ε4 status and may be involved in mediating the effect of *APOE* genotype, supporting a role for the presence of this lncRNA in disease risk and progression, although it is also feasible that Alzheimer’s disease pathology could drive intron-3 retention

## Discussion

The core aim of this study was to test the hypothesis that capturing human-lineage-specific regions of the genome could provide insights into neurological phenotypes and diseases in humans. We generated and used an annotation based on existing knowledge of sequence conservation and sequence constraint within humans, which we termed CNCRs. We used this annotation to prioritise genomic regions, genes and transcripts based on a high density of human-lineage-specific sequence as determined by our CNCR annotation. We demonstrated the utility of this approach by showing: the genomic regions we identified are enriched for SNP heritability for intelligence test performance and brain-related disorders; the genes we identified are enriched for neurologically relevant gene ontology terms and genes causing neurogenetic disorders and the existence of an intron-3 retaining transcript of *APOE*, the usage of which is correlated with Alzheimer’s disease pathology and *APOE*-ε4 status.

A major finding of this study is that CNCRs are enriched for regulatory, non-coding genomic regions. This is consistent with analyses performed by Ward and Kellis^14^, and highlights the potential functional importance of non-conserved and thus evolutionarily recent non-coding regions subject to constraint. Furthermore, these findings suggest that CNCRs could provide a means of prioritising and potentially aiding the assessment of non-coding variants, an area of significant interest, given that 88% of GWAS-derived disease-associated variants reside in non-coding regions of the genome^28^. We found evidence to support this view through heritability analyses for intelligence test performance, Parkinson’s disease, major depressive disorder and schizophrenia with SNP heritability not only enriched within CNCRs, but to a greater extent than would be expected using either conservation or constraint annotations alone. Considering heritability for intelligence test performance, this phenotype is already known to also be enriched within annotations of brain-specific tissue expression and among several regulatory biological gene sets^29^, including neurogenesis, central nervous system neuron differentiation and regulation of synapse structure or activity^28^. These findings support our hypothesis that CNCRs identify genomic regions of functional importance with relevance to human brain phenotypes.

Our analyses of CNCR density within genes are consistent with these findings, highlighting both non-coding genes and those implicated in neurologically relevant processes and diseases. Interestingly, CNCR annotation specifically highlighted lncRNAs as opposed to other non-coding RNAs. In particular, we observed a proportional increase in lncRNA enrichment with higher genic CNCR density, which could not be replicated using measures of sequence constraint or conservation alone. This observation is in keeping with previous studies that have shown most lncRNAs are tissue-specific with the highest proportion being specific to brain^30^ and highly relevant to neurodegenerative diseases^31^. Similarly, the enrichment for nervous system-related pathways within CNCRs, which is representative of recent purifying selection, is in keeping with the lowest proportion of positively selected genes being present in brain tissues from previous studies of mammalian organ development^32^. We also find enrichment of spinal cord-associated genes that may relate to the uniquely human monosynaptic corticomotoneuronal pathways implicated in human-specific dexterity and digital motor control^33,34^, the disruption of which may lead to amyotrophic lateral sclerosis^35^.

We noted that *APOE* was among the genes with the highest CNCR density across the genome and carried the highest CNCR density of all genes implicated in complex brain-relevant phenotypes (defined within the STOPGAP database^19^). Given that genetic variation within this gene and specifically *APOE*-ε4 status is not only the principal genetic risk factor for Alzheimer’s disease^36^ but also associated with risk for other neurodegenerative disorders, stroke and reduced lifespan^20^, this finding provides evidence for the value of CNCR annotation. We thus further studied *APOE* to validate our annotation. Within *APOE*, the CNCR annotation highlighted an intron-3 retention event of high coverage and CNCR density, not currently within annotation but which has been previously reported to be associated with neuronal regulation of *APOE*, with splicing out of the intron-3-containing mRNA following neuronal injury in neuronal cell lines and human *APOE* knock-in mouse models^21,23,24^. Using Sanger sequencing of cDNA derived from control human hippocampal tissue, we confirm the presence of an intron-3 retaining *APOE* transcript. We estimated the usage of the transcript from short-read RNA-sequencing data and found variable levels across different brain tissues within GTEx^25^ with the highest usage in the spinal cord, substantia nigra and hippocampus, reflecting central nervous system regions most susceptible to selective vulnerability in disease. Using human dorsolateral prefrontal cortex RNA-sequencing data, we found that the intron retention event was significantly more abundant in patients with Alzheimer’s disease than controls and in those with more severe Braak and Braak pathology and amyloid burden as characterised by CERAD pathology. Furthermore, we saw a dosage-dependent increase in the intron retention event with the *APOE*-ε4 allele that was independent of disease status. Although our findings do not elucidate the function of the intron-3 retention event, they are consistent with previous studies that have shown general increases in intron retention events as a feature of Alzheimer’s disease and ageing with implications for post-transcriptional regulation^37^. We propose that this novel transcript may be a means of regulating *APOE* in a disease state or could itself be driven by Alzheimer’s disease pathology.

Given that we use existing measures of constraint and conservation to identify CNCRs, this analysis is fundamentally limited by the quality of these data. While the constraint metrics we used were derived from high-depth sequencing, this is still restricted given the relatively high number of private genetic variants we each carry. In addition, analysis was limited to the high-confidence regions covering ~84% of the genome, amounting to 12.2% of all genes that remained unannotated with CDTS metrics^11^. Thus, on balance, it is difficult to predict the impact of these missing data on our findings. Similarly, our study of the relationship between CNCRs and known genomic features is limited by the annotation quality in existing databases. We have endeavoured to overcome some of these problems by creating a more detailed annotation combining both GENCODE and Ensembl data as used by di Iulio et al. in their work generating CDTS^11^. The SNP heritability estimates using stratified-linkage disequilibrium score regression (LDSC) analysis are limited by the quality of linkage disequilibrium (LD) information underpinning the heritability calculations^38^ and the sample size of the GWAS.

Despite these limitations, we have been able to demonstrate the utility of CNCRs specifically in the identification of functionally important non-coding regions of the genome, genes and transcripts. We find that CNCRs across all forms of analyses highlight the significance of human-lineage-specific sequences in the central nervous system and in the context of neurological phenotypes and diseases. We release our annotation of CNC scores and CNCRs via the online platform vizER (https://snca.atica.um.es/browser/app/vizER) to allow CNCRs to be viewed at a granular level. Thus, the CNCR annotation we generate has the potential to provide additional disease insights beyond those explored within this study and as we anticipate the release of increasing quantities of WGS data in humans will only improve in quality and value.

## Methods

### Generation of an annotation for the identification of CNCRs

We generated a combined annotation to capture information on intra-species constraint and inter-species conservation simultaneously, using CDTS together with phastCons20 scores (Fig. 1). The previously validated map of sequence constraint (http://www.hli-opendata.com/noncoding) generated using 7794 whole-genome sequences^11^ was used to assign a single CDTS score to each non-overlapping 10 bp region throughout the genome (build GRCh38, 248,925,226 bins). The phastCons20 score, which calculates the likelihood ratio of negative selection based on the total number of substitutions during evolution of an element between species^39^, was used as a measure of inter-species conservation (http://hgdownload.cse.ucsc.edu/goldenPath/hg38/phastCons20way/)^39^. PhastCons20 was used as it compares the human genome to the genomes of less divergent species (16 other primates and three mammals). For each 10 bp bin, we assigned the corresponding mean phastCons20 score. Bins without a conservation score due to insufficient species in the alignment were not considered (0.218% of the genome), nor did we consider bins without a CDTS score (16% of the genome, equating to 12.2% of all genes). We found that 10.9% of the unannotated regions of the genome were within the ENCODE list of problematic regions (https://github.com/Boyle-Lab/)^40^ with the remainder accounted for by incomplete sequencing from the 10,000 Genomes Project^10^. We recognise that this is a limitation of this study and of the previously reported analysis^11^. For the remaining 248,381,744 bins, we ranked both CDTS and mean phastCons20 scores across the whole genome such that the highest ranks represented the most constrained and conserved regions, respectively. We calculated the log_2_ ratio of the rank of constraint to the rank of conservation for each 10 bp bin (termed constrained, non-conserved score, CNC score). This resulted in scores with a distribution centred at 0 signifying no fold change between the ranks of the two metrics (Supplementary Fig. 1). Finally, we defined CNCRs as genomic regions that were first among the 12.5% most constrained, then with a CNC score of ≥1 (i.e. a twofold higher ranking in constraint than conservation). We used this definition for CNCRs throughout this study to capture regions that were among the most constrained, but less conserved genome.

### Investigating the relationship between CNCRs and existing annotation

To investigate the relationship between CNC scores for genomic regions and genomic features, we calculated the distribution of CNC scores across genomic features defined by GENCODE v.53^41^ and Ensembl v.92^42^. We restricted our analysis to the 12.5% most constrained regions only (31,115,616 ten bp bins) and segregated these regions into equally sized deciles ranked on the basis of CNC scores such that the highest decile (90–100 decile) represented a high CNC score containing the most constrained and least conserved sequences. Each 10 bp region was then assigned a single overlapping genomic feature. To avoid conflicts arising from overlapping GENCODE and Ensembl definitions, we preferentially assigned a single genomic feature to a given region by prioritising features as used by di Iulio et al.^11^ (described in Supplementary Table 1). In order to compare the enrichment of existing annotations within the proportions of the different genic regions, we used chi-squared test with Yate’s continuity correction, implemented in R v.3.6.1.

### Enrichment of common-SNP heritability in brain-related phenotypes for CNCRs

Stratified-LDSC was used to assess the enrichment of common-SNP heritability for a range of complex diseases and traits within our annotation^38,43^. Stratified-LDSC makes use of the increased likelihood of a causal relationship in a block of SNPs in LD to correct for confounding biases that include cryptic relatedness and population stratification in a polygenic trait^43^. Using established protocols (https://github.com/bulik/ldsc/wiki), we tested whether SNPs located within our annotation contributed significantly to SNP heritability after controlling for a range of other annotations described within the baseline mode (v.1.2). This analysis generates a coefficient *z*-score, from which we calculated a one-tailed coefficient *p* value. Stratified-LDSC regression analyses were also run to incorporate background SNPs defined as all SNPs in the genome that include a CDTS and phastCons20 annotation, to avoid overestimation of the contribution to SNP heritability. We assessed the annotation for SNP heritability enrichment in complex brain-related disorders and phenotypes of intelligence test performance^29^, Alzheimer’s disease^44^, Parkinson’s disease (excluding 23&Me participants)^45^, schizophrenia^46^ and major depressive disorder (excluding 23&Me participants)^47^ (Supplementary Table 2). We considered SNPs within CNCRs and its two constituent groups (Fig. 1) which fall either into constrained-only or non-conserved only annotations as defined respectively by: (i) CNCRs annotation: SNPs falling into CNCRs; (ii) constrained annotation: SNPs located within the 12.5% most constrained regions of the genome irrespective of conservation score and (iii) non-conserved annotation: SNPs located within relatively non-conserved genomic regions with a conservation rank determined by the rank of the first quartile phastCons20 score at a CNC score of 1 (rank ≤ 25,623,592) (irrespective of constraint score). We provided Bonferroni-corrected*p* values, which account for the number of annotation categories and GWASs tested (total of 15 conditions).

### Generation of a gene-based metric for CNCRs and gene set enrichment analysis

To generate a metric of human-specific constraint, which could be applied to a gene rather than a 10 bp region, we calculated the density of CNCRs within each gene, the length of which was defined by the transcription start and stop sites for that gene (GRCh38.v97). The CNCR density was defined as the proportion of the length of a gene containing CNCRs (Supplementary Fig. 1d). In this way, we were able to normalise for the effect of gene size on our metric. Therefore, the higher the gene density, the larger the proportion of the total length of the gene was covered by CNCRs.

In order to compare the relationship between the change in CNCR density and the proportion of a genic biotype (defined by Ensembl v.92), we used linear regression and applied FDR-corrected*p* values in R v.3.6.1.

We used g:ProfileR (R Package)^48^ for gene set enrichment analysis. We used the three sets of tested annotations incorporating genes that fell into CNCRs, constrained regions and non-conserved regions in the gene set enrichment analysis as previously described for LDSC annotation and as defined in Fig. 1. The background gene list in all analyses comprised 49,644 genes from all regions of the genome with a CDTS and phastCons20 annotation. The correction method was set to g:SCS to account for multiple testing^48^. We used REViGO^49^ to summarise the significant GO terms, and to derive the term frequency, which is a measure of GO term specificity.

To further characterise CNCR density within genes associated with disease, we first studied phenotype relationships of all Mendelian genes within the OMIM catalogue (http://api.omim.org)^18^. We compared the CNCR density of all neurologically relevant OMIM genes to all genes within CNCR annotation. Secondly, in order to investigate the CNCR density within genes associated with complex disorders, we used the STOPGAP database, a catalogue of human genetic associations mapped to effector gene candidates derived from 4684 GWASs^19^. We selected for genes associated with SNPs that surpassed a genome-wide significant *p* value of 5 × 10^−8^ and which fulfilled medical subject heading for associated neurological/behavioural diseases. We used these sets to identify potential genes of interest associated with brain-related disorders which carry a high CNCR density.

### Sequencing of *APOE* transcripts in human brain

Focussing on a region with high CNCR density identified within *APOE* from the preceding analyses, we used Sanger sequencing of cDNA reverse transcribed from pooled human hippocampus poly-A-selected RNA (Takara/Clontech 636165) to support the presence of the intron-3 retention event identified within *APOE* (GRCh38: chr19:44907952-44908531). For the reverse transcription, we used 500 ng of input RNA, with 10 mM dNTPs (NEB N0447S), VN primers and strand-switching primers (Oxford Nanopore Technologies SQK-DCS109), 40 units of RNaseOUT inhibitor (Life Technologies 10777019) and 200 units of Maxima H Minus reverse transcriptase with 5X reverse transcription buffer (Thermo Fisher EP0751). PCR amplification of the cDNA was performed using primer pairs designed to span across intron-3 and exon 4 (P2-4) and intron-3 alone (P5) of *APOE* (ENST00000252486.9) (Supplementary Table 3). PCR was performed using Taq DNA polymerase with Q-solution (Qiagen) and enzymatic clean-up of PCR products was performed using Exonuclease I (Thermo Fisher Scientific) and FastAP thermosensitive alkaline phosphatase (Thermo Fisher Scientific). Sanger sequencing was performed using the BigDye terminator kit (Applied Biosystems) and sequence reactions were run on ABI PRISM 3730xl sequencing apparatus (Applied Biosystems). Electropherograms were viewed and sequences were exported using Sequencher 5.4.6 (Gene Codes). Sequences were aligned against the human genome (hg38) using BLAT and visually inspected for confirmation of validation.

To reduce the risk of gDNA contamination, the human hippocampus poly-A-selected RNA (Takara/Clontech 636165) had undergone selection by two rounds of oligo(dT)-cellulose columns. Furthermore, the Maxima H Minus reverse transcriptase buffer contains a double-strand-specific DNase to specifically remove gDNA. Lastly, we used poly-A selected RNA sample as a no-reverse transcriptase control in comparison with cDNA to show that there is no contamination with gDNA. A total of 100 ng poly-A-selected RNA and 100 ng cDNA was used for each reaction in a total volume of 10 µl using the same PCR conditions. PCR amplification of cDNA and RNA was performed using primer pair P2 using Taq DNA polymerase (Qiagen protocol) with 30 s of denaturation and 30 s of annealing at 57 °C.

### Quantifying differences in intron-3 retention between different species

In order to investigate species differences in the human *APOE* intron-3 retention event identified with chimpanzees, we leveraged existing bulk RNA-sequencing data derived from chimpanzee and human hippocampus reported in Khrameeva et al.^50^. We used data from the hippocampus and downloaded FASTQ files (NCBI Gene Expression Omnibus; https://www.ncbi.nlm.nih.gov/geo/, accession number GSE127898) from the three available human samples (SAMN11165674, SAMN11165673, SAMN11165737) and three available chimpanzee samples (SAMN11166008, SAMN11165613, SAMN11165949). All bulk RNA-sequencing FASTQ files were aligned using STAR^51^ with a reference index generated for the relevant species. Given the small number of samples, direct visualisation of the aligned BAM files in Integrative Genome Viewer^52^ across *APOE* was carried out. Furthermore, for all samples, the total coverage of intron-3 was normalised for the length of intron-3 in each species and also for the total *APOE* coverage accounting for sequencing depth and *APOE* expression differences to allow cross-species and cross-sample comparisons. The co-ordinates for the regions were taken from Ensembl for human GRCh38 and chimpanzee Pan_tro_3.0.

### Analysis of public RNA-sequencing data

We used publicly available short-read RNA-sequencing data from human brain post-mortem samples provided by GTEx v.7.1^25^ and the ROSMAP Study^53^ and to quantify the intron-3 retention event in *APOE* highlighted by our analysis. For GTEx data, we used pre-aligned files available from recount2 (https://jhubiostatistics.shinyapps.io/recount/)^54^. Both studies within ROSMAP are longitudinal clinicopathological cohort studies of aging and/or Alzheimer’s disease. We downloaded BAM files for ROSMAP bulk RNA-sequencing data from the Synapse repository (https://www.synapse.org/#!Synapse:syn4164376) for analysis. To quantify the intron-3 retention event, we calculated the coverage of intron-3 expression normalised for the coverage across the entire *APOE* gene, as defined by the transcription start and end sites. To quantify splicing of intron-3, we calculated the number of exon 3 to exon 4 junction reads (defined as reads mapping with a gapped alignment), normalised for all *APOE* junction reads detected and currently within annotation. We used a ratio of the normalised coverage to normalised junction count over intron-3 as an estimate of the proportional use of the intron-3-retaining transcript, such that a high ratio is associated with a higher usage of intron retention within both GTEx and ROSMAP data. Normalisation of the intron-3 event for *APOE* gene expression (directly proportional to the canonical transcripts) was used to show independent effects of the intron-3 event from the canonical transcripts. Comparisons between the two groups were performed by comparing the mean values of this normalised measure using Wilcoxon rank sum test, taking two-tailed*p* values < 0.05 to be significant. Based on existing ROSMAP results^55^ and principal component analysis of fragments per kilobase million data, we incorporated covariates to account for the effect of batch, RNA integrity number, post-mortem interval, study index, ethnicity, age at death and sex on estimates of intron-3-retaining transcript usage. Using the resulting linear regression model, we compared the intron-3 retention normalised coverage to junction ratio across clinical disease states, pathological states and *APOE*-ɛ4 status in 634 post-mortem brain samples.

### Reporting summary

Further information on research design is available in the Nature Research Reporting Summary linked to this article.

## Supplementary information

Supplementary Information

Reporting Summary

## Data Availability

We release our annotation of CNC score as an interactive visualisable track via online platform vizER: (https://snca.atica.um.es/browser/app/vizER) and provide a publicly downloadable table of CNCR density for genes within our annotation (under the “Download” Tab). Publicly available datasets used are: CDTS metrics: http://www.hli-opendata.com/noncoding. phastCons20 metrics: http://hgdownload.cse.ucsc.edu/goldenPath/hg38/phastCons20way/. ROSMAP studies BAM files: https://www.synapse.org/#!Synapse:syn4164376. OMIM API: http://api.omim.org. STOPGAP database: https://github.com/StatGenPRD/STOPGAP/blob/master/STOPGAP_data/stopgap.bestld.RData. GTEx portal: https://www.gtexportal.org/home/datasets. Ensembl v92: https://www.ensembl.org/index.html. GENCODE: https://www.gencodegenes.org/pages/data_access.html. ENCODE list of problematic regions: https://github.com/Boyle-Lab/. Chimpanzee and human bulk RNA-sequencing data: NCBI Gene Expression Omnibus; https://www.ncbi.nlm.nih.gov/geo/, accession number GSE127898). Source Data are provided with this paper.

## Source data

Source Data
