# Supplementary material for: Human-lineage-specific genomic elements are associated with neurodegenerative disease and APOE transcript usage
Source: Nat Commun. 2021 Apr 6;12:2076. doi: 10.1038/s41467-021-22262-5 (PMC8024253; doi:10.1038/s41467-021-22262-5)
Supplement: Supplementary file 1 — Supplementary Information [file 41467_2021_22262_MOESM1_ESM.pdf]

Supplementary Figures

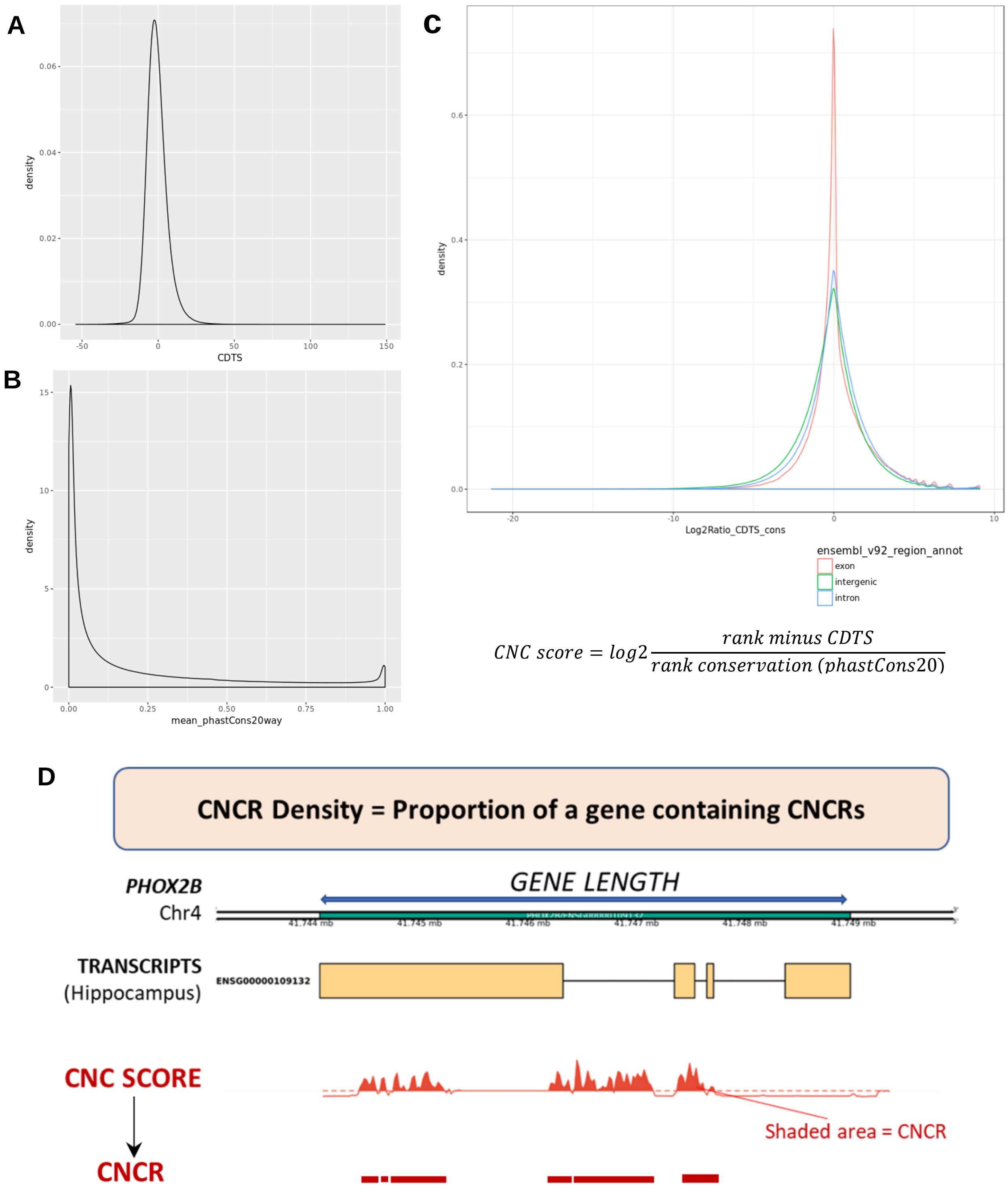

Supplementary Figure 1. Kernal density plots of annotation metrics and explanation of constrained, non-conserved regions (CNCR) density metric. Panel **a** depicts density plot of genomic constraint (context dependent tolerance score (CDTS): a lower CDTS represents more constrained data). Panel **b** shows the density distribution of the mean phastCons20 scores per 10bp bin with a score of 1 showing higher conservation than 0. Panel **c** shows the distribution of $\log_2$ ratio (constrained, non-conserved (CNC) score) of the reverse ranked CDTS (so a higher rank pertains to higher constraint but lower CDTS) and ranked phastCons20 scores, partitioned by regions of exon,

intron and intergenic as defined by Ensembl v.92. Panel **d** provides a schematic explanation for how the CNCR density is calculated, using *PHOX2B* gene as an example. The CNCR density is calculated using the number of CNCRs within a gene (shown in the shaded area in the figure from the CNC track and also shown as a separate CNCR track where each bar represents a region fulfilling criteria for CNCR), divided by the total gene length (shown in the top track of the figure).

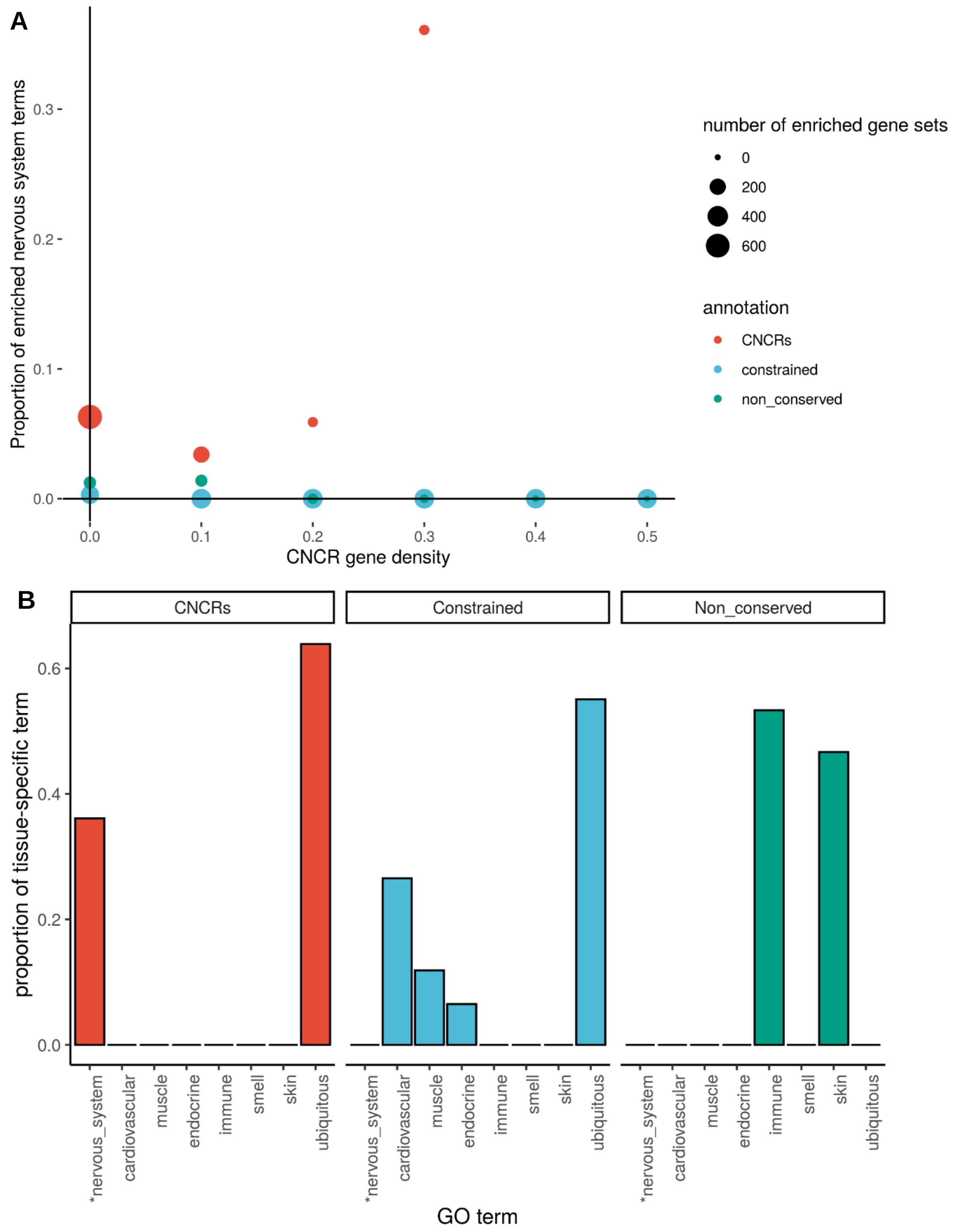

Supplementary Figure 2. Proportion of enriched neurologically-related gene ontology (GO) terms in the gene set analysis compared between the annotation of interest (constrained, non-conserved regions: CNCRs) and the comparator annotation sets (a). Proportion of neurologically-related GO terms at CNCR density of 0.3 and above (b).

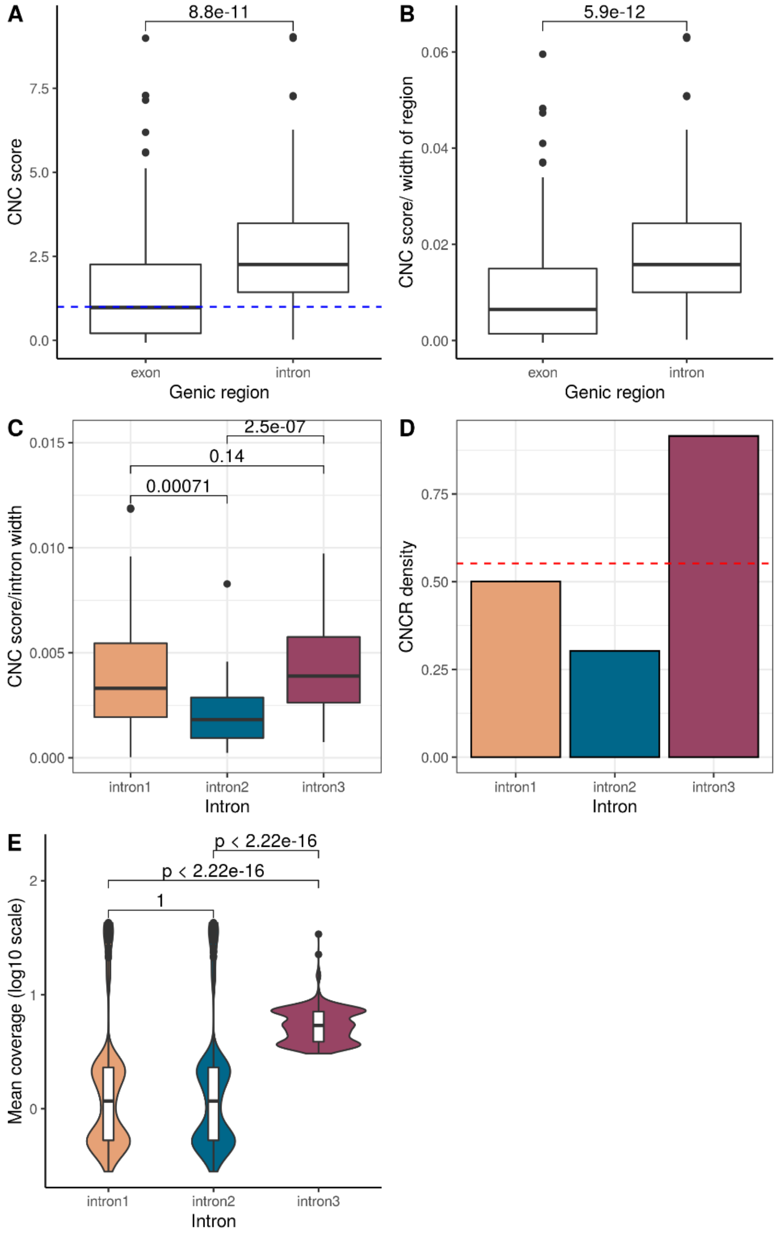

Supplementary Figure 3. Justification for further intragenic analysis of the intron-3 retention event in *APOE*. Panel A shows boxplot comparing constrained, non-conserved (CNC) scores across all *APOE* exonic and intronic regions with the blue dashed line showing a CNC score of 1 that is used as the cut-off for defining constrained, non-conserved regions (CNCRs). The number above the square brackets denotes the Wilcoxon rank sum test p-value comparing the two groups. Panel B shows the CNC score normalised for the width of each genic region. The number above the square brackets denotes the Wilcoxon rank sum test p-value comparing the two groups. Panel C shows the CNC score normalised for width of each individual intron across the three introns of *APOE* ENST00000252486. The number above the square brackets denotes the Wilcoxon rank sum test p-value comparing the two groups. Panel D shows the local CNCR density for each intron within *APOE* ENST00000252486. The red dashed line shows the CNCR density across the entire *APOE* gene. Panel E shows the $\log_{10}$ mean coverage of the three different *APOE* introns within GTEx hippocampal tissue. The number above the square brackets denotes the Wilcoxon rank sum test p-value comparing each of the two groups.

A

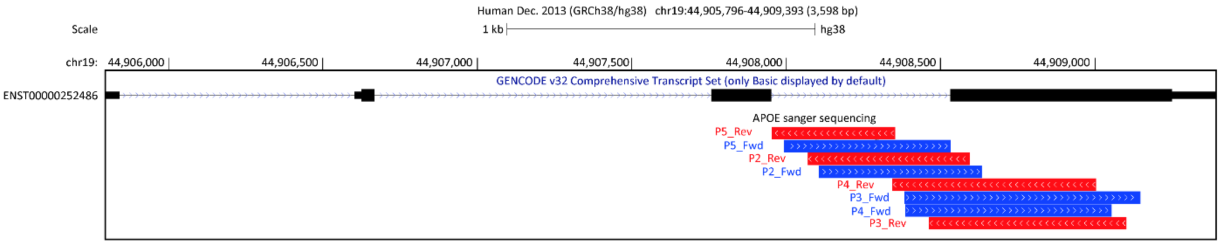

B

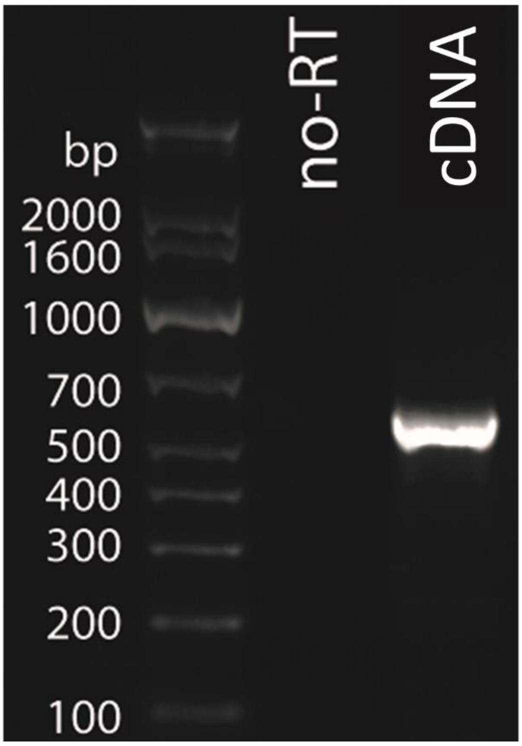

Supplementary Figure 4. Panel a: Sanger sequencing of human hippocampus cDNA using targeted primers within *APOE*, aligned to hg38. Panel b: Gel electrophoresis results of PCR amplification using P2 primer pair for a no-reverse transcriptase (no-RT) control (poly-A selected RNA) (lane labelled “no-RT”) and for cDNA (lane labelled “cDNA”). Four different primer pairs (as listed in Supplementary Table 3) were used once each.

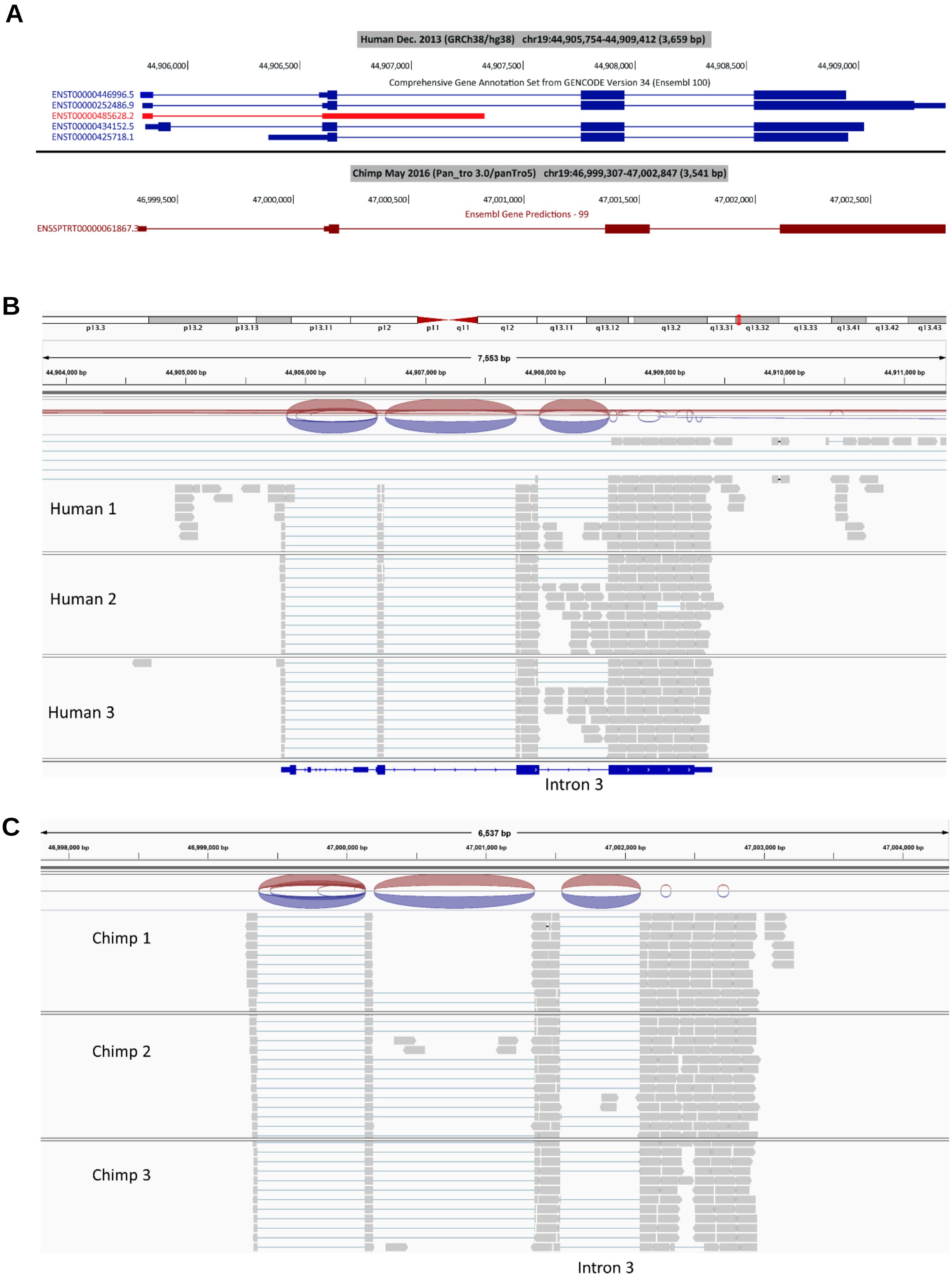

Supplementary Figure 5. (**a**) Schematic representation of annotated transcripts for both human and chimpanzee in Ensembl GRCh38 and pan_tro 3.0. (**b**) Selected representations of the alignments of three human hippocampal bulk RNA-sequencing reads from Khrameeva *et al.*[1] across *APOE* with reads spanning over intron 3 present taken from Integrative Genome Viewer. (**c**) Selected representations of the alignments of three chimpanzee hippocampal bulk RNA-sequencing reads across *APOE* with occasional reads spanning over intron 3.

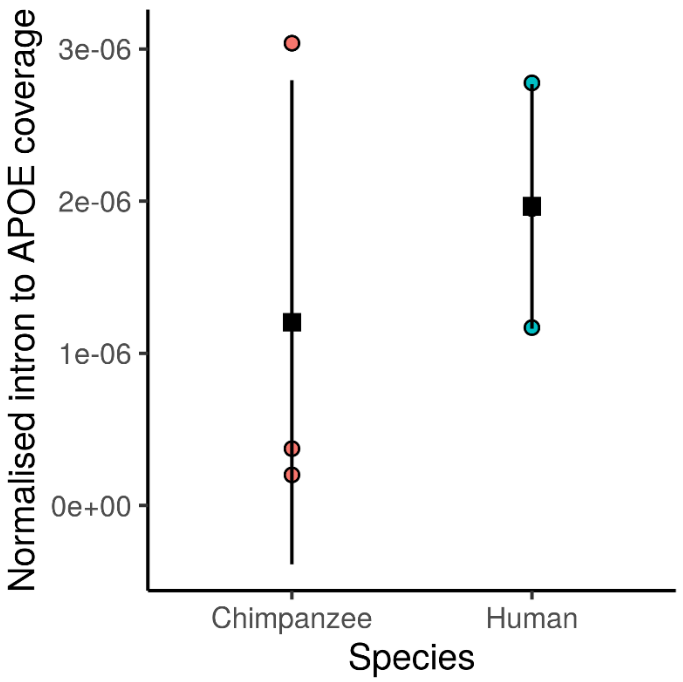

Supplementary Figure 6. Comparison of the intron-3 coverage: *APOE* coverage normalised for the intronic length of intron-3 across all three human and all three chimpanzee samples. Values for the samples are represented using the round points. The black square represents the mean of the datapoints with the black vertical lines extending to the mean ± standard error of the mean. There were no statistically significant differences between the mean intron retention event between humans and chimpanzees (Wilcoxon rank sum p-value = 0.7).

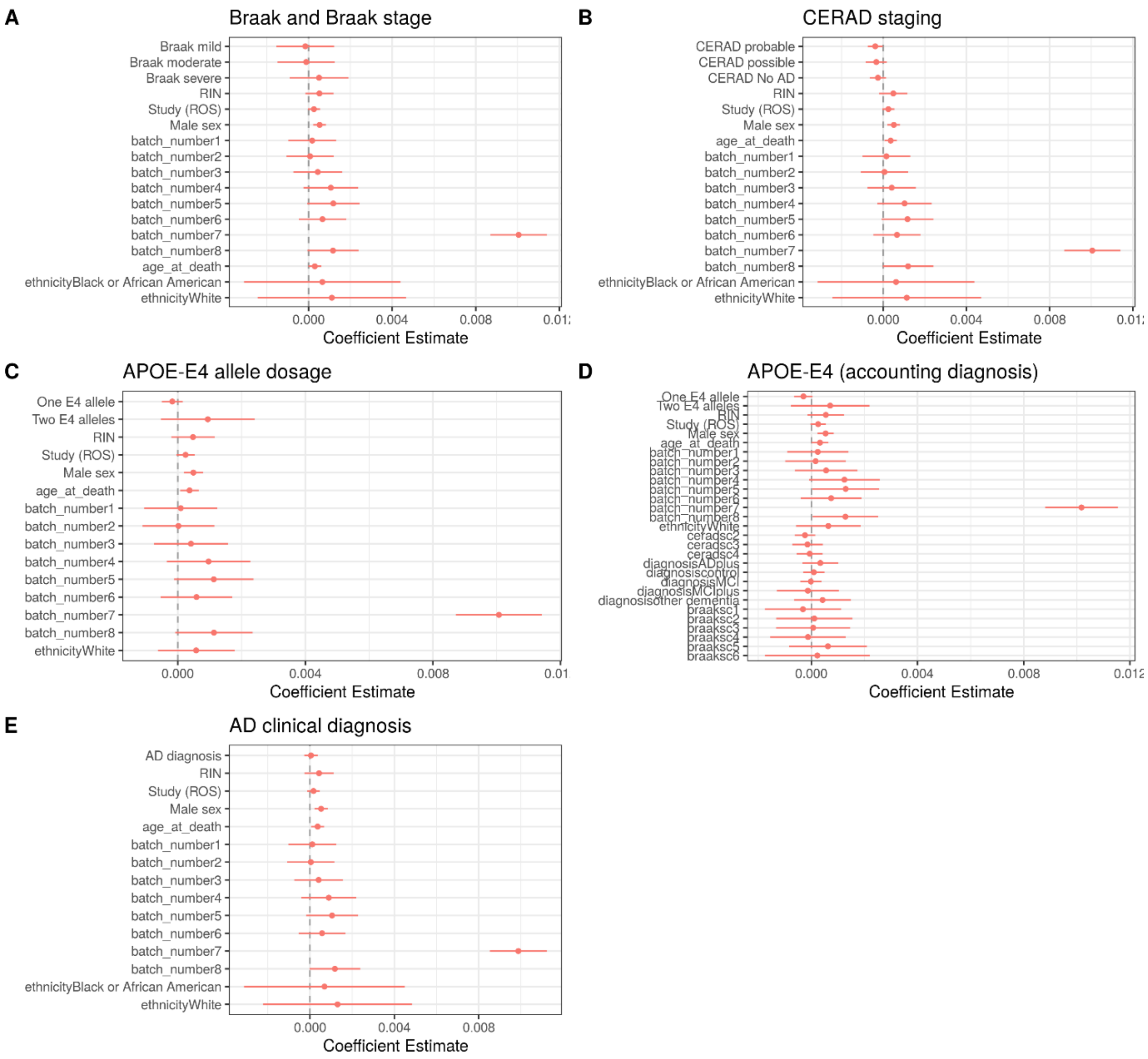

Supplementary Figure 7: Coefficient effect estimates for covariates (red dot) +/- 95% confidence interval (horizontal line) from ROSMAP bulk RNA-sequencing studies of dorsolateral frontal cortex tissue using linear regression models to interrogate the relationship between *APOE* intron-3 retention usage and Braak and Braak stage (Panel A); CERAD staging (Panel B); *APOE*-ε4 allele dosage with conventional ROSMAP covariates (Panel C); *APOE*-ε4 allele dosage with conventional ROSMAP covariates and additional covariates incorporated for Alzheimer’s disease diagnosis, CERAD and Braak and Braak staging (Panel D); and clinical Alzheimer’s disease diagnosis (Panel E).

Supplementary Tables

Supplementary Table 1. Annotation priority order for genomic feature. Genomic features are based on both Gencode and Ensembl. A priority order for annotation with a genomic feature is assigned to avoid conflict with overlapping features as used in di Iulio *et al*.[2] The number of 10bp bins for each genomic feature across the genome is also shown in the table.

**Annotation priority order**	**Genomic feature**	**Number of 10bp bins**	**Description**
1	Exon PCCDS	1,453,269	Exon, protein-coding sequence
2	Exon NCRNA	1,156,726	Exon, non-coding RNA, e.g. lincRNA
3	Exon PCUTR	892,210	Exon, protein-coding UTR
4	Promoter	820,321	Promoter
5	Promoter Flanking	1,074,641	Cluster with promoters or distal cis-regulatory elements
6	Enhancer	251,636	Enhancer
7	Intron, cis	108,670	Introns located in genes <10bp from splice-site
8	Intron, trans	15,204,447	Introns located in genes >10bp from splice-site
9	Intergenic	689,419	Not annotated in GenCode/ Ensembl
10	H3K9me3	2,082,553	Only overlap with H3K9me3
11	H3K27me3	777,409	Only overlap with H3K27me3
12	Multiple histones	5,199,455	Overlap with a combination of histone marks
13	Other	1,404,860	Includes open chromatin and unannotated features

Supplementary Table 2. Genome-wide association studies used in the stratified linkage-disequilibrium score regression (s-LDSC) analysis. The GWAS for Parkinson's disease and major depressive disorder do not incorporate 23&Me data.

Phenotype	Author, Year, Reference	n case
Intelligence test	Savage, 2018[24]	269,858
Alzheimer's disease (AD)	Jansen, 2019[25]	71,880
Parkinson's disease (PD)	Nalls, 2019 (excluding 23&Me data)[26]	33,674
Major depressive disorder (MDD)	Wray, 2018 (excluding 23&Me data)[28]	59,851
Schizophrenia (SCZ)	Pardiñas, 2018[27]	40,675

Supplementary Table 3. Primer positions and sequences used to validate the *APOE* intron-3 retention event.

Primer name	5' – 3' sequence	Strand	Chr: Start-End (hg38)
**P2_Fwd**	GGTTCTAGCTTCCTCTTCCC	+	19:44908064-44908083
**P2_Rev**	CGCCTGCAGCTCCTTGGACAG	-	19:44908627-44908647
**P3_Fwd**	CCTAGCTCCTTCTTCGTCTC	+	19:44908337-44908356
**P3_Rev**	CTCGAACCAGCTCTTGAGG	-	19:44909130-44909148
**P4_Fwd**	CCTTCTTCGTCTCTGCCTC	+	19:44908344-44908362
**P4_Rev**	CTGCTCCTTCACCTCGTC	-	19:44909037-44909055
**P5_Fwd**	GTGAGTGTCCCCATCCTGG	+	19:44907953-4490771
**P5_Rev**	CTGCGGCCGAGAGGGCGGGAG	-	19:44908512-44908532

Supplementary Table 4. Results for heritability, enrichment, and regression coefficient from stratified linkage-disequilibrium score regression (s-LDSC) analysis. The coefficient p-values are one-sided p-values calculated from the coefficient Z-score.

Annotation	GWAS	Proportion SNPs	Proportion heritability	Enrichment	Enrichment p-value	Regression Coefficient	Coefficient Z-score	Z- score -log P-value
**CNCR**	**Intelligence 2018**	0.031	0.339	11.044	5.12E-20	2.96E-07	10.059	23.378
**Constrained**		0.055	0.441	8.066	3.20E-21	1.85E-07	9.413	20.618
**Non-conserved**		0.126	0.330	2.628	1.32E-05	6.28E-08	5.125	6.828
**CNCR**	**AD 2019**	0.031	0.398	12.974	0.00987	1.89E-08	1.961	1.603
**Constrained**		0.055	0.532	9.733	0.00196	1.12E-08	1.965	1.607
**Non-conserved**		0.126	-0.341	-2.713	0.216	-8.51E-09	-1.585	0.025
**CNCR**	**PD 2019 (ex.23&Me)**	0.031	0.334	10.884	0.00193	2.57E-08	2.767	2.548
**Constrained**		0.055	0.367	6.715	0.00881	1.32E-08	2.080	1.727
**Non-conserved**		0.126	0.149	1.191	0.857	1.28E-10	0.037	0.314
**CNCR**	**MDD 2018 (ex.23&Me)**	0.031	0.330	10.755	1.39E-07	1.13E-07	5.422	7.530
**Constrained**		0.055	0.404	7.379	1.51E-08	6.29E-08	4.941	6.410
**Non-conserved**		0.126	0.433	3.446	5.02E-04	3.84E-08	3.908	4.333
**CNCR**	**SCZ 2018**	0.031	0.339	11.033	2.50E-16	6.53E-07	8.829	18.279
**Constrained**		0.055	0.425	7.772	2.19E-17	4.04E-07	8.456	16.860
**Non-conserved**		0.126	0.309	2.461	8.75E-04	1.18E-07	3.576	3.759

Supplementary Table 5. Significantly enriched nervous system-related gene ontology (GO) terms for constrained, non-conserved regions (CNCRs) at density of 0.3. P-value relates to the p-value for enrichment calculated using g:Profiler and its own g:SCS correction method[3].

GO ID	GO term description	P-value
GO:0048663	neuron fate commitment	5.46E-07
GO:0048665	neuron fate specification	0.0012
GO:0021510	spinal cord development	0.00129
GO:0021517	ventral spinal cord development	0.00175
GO:0021515	cell differentiation in spinal cord	3.64E-07
GO:0021953	central nervous system neuron differentiation	7.44E-05
GO:0021522	spinal cord motor neuron differentiation	3.48E-04
GO:0021520	spinal cord motor neuron cell fate specification	0.0479
GO:0021527	spinal cord association neuron differentiation	0.00533
GO:0021871	forebrain regionalization	7.91E-05
GO:0021978	telencephalon regionalization	0.00313
GO:0030902	hindbrain development	0.0337
GO:0021536	diencephalon development	0.045

Supplementary Table 6. Wilcoxon rank sum test pairwise comparison p-values for the normalised intron-3 coverage to junction ratio between different GTEx brain tissues. Significant p-values (<0.05) are highlighted in orange. The three brain regions with the highest normalised intron-3 coverage to junction ratio, namely the spinal cord, substantia nigra and hippocampus, are the only three regions in which the ratio is statistically significantly higher than all regions that have a normalised ratio lower than the median normalised ratio across all brain regions. These regions are the nucleus accumbens, putamen, anterior cingulate cortex, frontal cortex and cerebellar hemisphere.

Cerebellar Hemisphere											
Frontal Cortex	0.85										
Anterior cingulate cortex	0.29	0.46									
Putamen	0.04	0.094	0.38								
Nucleus accumbens	0.0018	0.0064	0.081	0.58							
Amygdala	0.00019	0.0013	0.0093	0.087	0.2						
Caudate	5.90E-05	0.00044	0.0092	0.13	0.29	0.64					
Hypothalamus	0.00097	0.004	0.027	0.15	0.29	0.8	0.89				
Hippocampus	4.10E-07	3.90E-06	0.00024	0.0064	0.014	0.47	0.19	0.32			
Substantia nigra	1.80E-06	2.50E-05	0.0004	0.0096	0.015	0.39	0.15	0.29	0.85		
Spinal cord	1.10E-14	9.00E-14	9.00E-12	3.00E-10	3.10E-10	9.00E-07	7.60E-09	1.10E-07	1.20E-06	1.20E-05	
Pair-wise comparisons p-value (Wilcoxon rank sum test)	Cerebellar Hemisphere	Frontal Cortex	Anterior cingulate cortex	Putamen	Nucleus accumbens	Amygdala	Caudate	Hypothalamus	Hippocampus	Substantia nigra	Spinal cord
	Ratio lower than median ratio across all brain tissues					Ratio higher than median ratio across all brain tissues					

Supplementary References

1 Khrameeva, E. *et al.* Single-cell-resolution transcriptome map of human, chimpanzee, bonobo, and macaque brains. *Genome research* **30**, 776-789, doi:10.1101/gr.256958.119 (2020).

2 di Iulio, J. *et al.* The human noncoding genome defined by genetic diversity. *Nature genetics* **50**, 333-337, doi:10.1038/s41588-018-0062-7 (2018).

3 Reimand, J. *et al.* g:Profiler-a web server for functional interpretation of gene lists (2016 update). *Nucleic Acids Res* **44**, W83-89, doi:10.1093/nar/gkw199 (2016).
