# Supplementary material for: Human-lineage-specific genomic elements are associated with neurodegenerative disease and APOE transcript usage
Source: Nat Commun. 2021 Apr 6;12:2076. doi: 10.1038/s41467-021-22262-5 (PMC8024253; doi:10.1038/s41467-021-22262-5)
Supplement: Supplementary file 2 — Reporting Summary [file 41467_2021_22262_MOESM2_ESM.pdf]

nature research

Corresponding author(s): Dr Mina Ryten

Last updated by author(s): Dec 15, 2020

Reporting Summary

Nature Research wishes to improve the reproducibility of the work that we publish. This form provides structure for consistency and transparency in reporting. For further information on Nature Research policies, see our Editorial Policies and the Editorial Policy Checklist.

Statistics

For all statistical analyses, confirm that the following items are present in the figure legend, table legend, main text, or Methods section.

n/a	Confirmed	
[ ]	[x]	The exact sample size (*n*) for each experimental group/condition, given as a discrete number and unit of measurement
[x]	[ ]	A statement on whether measurements were taken from distinct samples or whether the same sample was measured repeatedly
[ ]	[x]	The statistical test(s) used AND whether they are one- or two-sided<br>*Only common tests should be described solely by name; describe more complex techniques in the Methods section.*
[ ]	[x]	A description of all covariates tested
[ ]	[x]	A description of any assumptions or corrections, such as tests of normality and adjustment for multiple comparisons
[ ]	[x]	A full description of the statistical parameters including central tendency (e.g. means) or other basic estimates (e.g. regression coefficient) AND variation (e.g. standard deviation) or associated estimates of uncertainty (e.g. confidence intervals)
[ ]	[x]	For null hypothesis testing, the test statistic (e.g. *F*, *t*, *r*) with confidence intervals, effect sizes, degrees of freedom and *P* value noted<br>*Give P values as exact values whenever suitable.*
[x]	[ ]	For Bayesian analysis, information on the choice of priors and Markov chain Monte Carlo settings
[x]	[ ]	For hierarchical and complex designs, identification of the appropriate level for tests and full reporting of outcomes
[ ]	[x]	Estimates of effect sizes (e.g. Cohen's *d*, Pearson's *r*), indicating how they were calculated

Our web collection on statistics for biologists contains articles on many of the points above.

Software and code

Policy information about availability of computer code

Data collection	We did not use unreported custom computer code or algorithm to generate results.<br>Recount2: https://jhubiostatistics.shinyapps.io/recount/<br>bioMart v2.34.2
Data analysis	R version 3.6.1<br>derfinder v1.14.0<br>Gviz v1.22.3<br>LDSC Regression: https://github.com/bulik/ldsc/wiki<br>g:ProfileR

For manuscripts utilizing custom algorithms or software that are central to the research but not yet described in published literature, software must be made available to editors and reviewers. We strongly encourage code deposition in a community repository (e.g. GitHub). See the Nature Research guidelines for submitting code & software for further information.

Data

Policy information about availability of data

All manuscripts must include a data availability statement. This statement should provide the following information, where applicable:

- Accession codes, unique identifiers, or web links for publicly available datasets
- A list of figures that have associated raw data
- A description of any restrictions on data availability

Data Availability: We release our annotation of CNC score as an interactive visualisable track via online platform vizER: (https://snca.atica.um.es/browser/app/vizER)

and provide a publicly-downloadable table of CNCR density for genes within our annotation (under the "Download" Tab).
Publicly-available datasets used are:
CDTS metrics: http://www.hli-opendata.com/noncoding
phastCons20 metrics: http://hgdownload.cse.ucsc.edu/goldenPath/hg38/phastCons20way/
ROSMAP Studies BAM files: https://www.synapse.org/#!Synapse:syn4164376
OMIM API: http://api.omim.org
STOPGAP database: https://github.com/StatGenPRD/STOPGAP/blob/master/STOPGAP_data/stopgap.bestld.RData
GTEx Portal - https://www.gtexportal.org/home/datasets
Ensembl v92 - https://www.ensembl.org/index.html
GENCODE: https://www.gencodegenes.org/pages/data_access.html
ENCODE list of problematic regions: https://github.com/Boyle-Lab/
Chimpanzee and human bulk RNA-sequencing data: NCBI Gene Expression Omnibus; https://www.ncbi.nlm.nih.gov/geo/, accession number GSE127898)

Field-specific reporting

Please select the one below that is the best fit for your research. If you are not sure, read the appropriate sections before making your selection.

[x] Life sciences   [ ] Behavioural & social sciences   [ ] Ecological, evolutionary & environmental sciences

For a reference copy of the document with all sections, see nature.com/documents/nr-reporting-summary-flat.pdf

Life sciences study design

All studies must disclose on these points even when the disclosure is negative.

Sample size	No sample size calculation was performed given that we re-analysed existing Religious Orders Study and Memory and Aging Project (ROSMAP) Study RNA-sequencing data. This large dataset of post-mortem brain transcriptomic data has been used in a large number of publications demonstrating its considerable power to detect gene expression changes across disease states and levels of neuropathology (Ng et al. Nature Neuroscience 20, 1418-1426 (2017)).
Data exclusions	No data were excluded within the analyses of the ROSMAP data.
Replication	The ROSMAP RNA-sequencing data is distinguished by its size and the extent of meta-data available regarding the post-mortem brain samples of which it is composed, making the identification of a suitable dataset for replication challenging.
Randomization	This is not relevant to our analysis given that the ROSMAP RNA-sequencing dataset used for one aspect of this manuscript is an existing dataset which is based on a cohort design for collection and analysis of post-mortem human brain tissue. Therefore, there is no means of retrospectively randomizing.
Blinding	This is not relevant to our analysis given that the ROSMAP RNA-sequencing dataset used for one aspect of this manuscript is based on a cohort design for collection and analysis of post-mortem human brain tissue. Therefore, there is no means of retrospectively randomizing.

Reporting for specific materials, systems and methods

We require information from authors about some types of materials, experimental systems and methods used in many studies. Here, indicate whether each material, system or method listed is relevant to your study. If you are not sure if a list item applies to your research, read the appropriate section before selecting a response.

Materials & experimental systems

n/a	Involved in the study	
[x]	[ ]	Antibodies
[x]	[ ]	Eukaryotic cell lines
[x]	[ ]	Palaeontology and archaeology
[x]	[ ]	Animals and other organisms
[ ]	[x]	Human research participants
[x]	[ ]	Clinical data
[x]	[ ]	Dual use research of concern

Methods

n/a	Involved in the study	
[x]	[ ]	ChIP-seq
[x]	[ ]	Flow cytometry
[x]	[ ]	MRI-based neuroimaging

Human research participants

Policy information about studies involving human research participants

Population characteristics	We used publicly-available RNAsequencing data from participants recruited to the ROSMAP Study taken from https://www.synapse.org/#!Synapse:syn4164376. For the analysis, we incorporated covariates to account for the effect of batch, RNA integrity number (RIN), postmortem interval (PMI), study index, ethnicity, age at death and sex based on existing ROSMAP results (Ng et al. Nature Neuroscience 20, 1418-1426 (2017)).
Recruitment	Recruited by ROSMAP studies (study design) as previously published by Bennett et al. Current Alzheimer research 9, 628-645 (2012).
Ethics oversight	Approved as per ethics application within ROSMAP studies.

Note that full information on the approval of the study protocol must also be provided in the manuscript.
